# Activity of immunoproteasome inhibitor ONX-0914 in acute lymphoblastic leukemia expressing MLL–AF4 fusion protein

**DOI:** 10.1038/s41598-021-90451-9

**Published:** 2021-05-25

**Authors:** Tyler W. Jenkins, Sondra L. Downey-Kopyscinski, Jennifer L. Fields, Gilbert J. Rahme, William C. Colley, Mark A. Israel, Andrey V. Maksimenko, Steven N. Fiering, Alexei F. Kisselev

**Affiliations:** 1 Department of Drug Discovery and Development, Harrison School of Pharmacy, Auburn University, PRB, 720 S. Donahue Dr., Auburn, AL 36849 USA; 2 Norris Cotton Cancer Center, Geisel School of Medicine, Dartmouth College, Lebanon, NH USA; 3 Department of Molecular and Systems Biology, Geisel School of Medicine, Dartmouth College, Lebanon, NH USA; 4 Department of Microbiology and Immunology, Geisel School of Medicine, Dartmouth College, Lebanon, NH USA; 5 Present Address: SLDK - Rancho Biosciences, San Diego, CA USA; 6 Present Address: GJR- Massachusetts General Hospital, Harvard Medical School, Boston, MA USA; 7 Present Address: Broad Institute of Harvard and MIT, Cambridge, MA USA; 8 Present Address: WCC - ScribeAmerica, Huntsville Hospital, Huntsville, AL USA; 9 Present Address: MAI- Israel Cancer Research Fund, New York, NY USA

**Keywords:** Targeted therapies, Acute lymphocytic leukaemia

## Abstract

Proteasome inhibitors bortezomib and carfilzomib are approved for the treatment of multiple myeloma and mantle cell lymphoma and have demonstrated clinical efficacy for the treatment of acute lymphoblastic leukemia (ALL). The t(4;11)(q21;q23) chromosomal translocation that leads to the expression of MLL–AF4 fusion protein and confers a poor prognosis, is the major cause of infant ALL. This translocation sensitizes tumor cells to proteasome inhibitors, but toxicities of bortezomib and carfilzomib may limit their use in pediatric patients. Many of these toxicities are caused by on-target inhibition of proteasomes in non-lymphoid tissues (e.g., heart muscle, gut, testicles). We found that MLL–AF4 cells express high levels of lymphoid tissue-specific immunoproteasomes and are sensitive to pharmacologically relevant concentrations of specific immunoproteasome inhibitor ONX-0914, even in the presence of stromal cells. Inhibition of multiple active sites of the immunoproteasomes was required to achieve cytotoxicity against ALL. ONX-0914, an inhibitor of LMP7 (ß5i) and LMP2 (ß1i) sites of the immunoproteasome, and LU-102, inhibitor of proteasome ß2 sites, exhibited synergistic cytotoxicity. Treatment with ONX-0914 significantly delayed the growth of orthotopic ALL xenograft tumors in mice. T-cell ALL lines were also sensitive to pharmacologically relevant concentrations of ONX-0914. This study provides a strong rationale for testing clinical stage immunoproteasome inhibitors KZ-616 and M3258 in ALL.

## Introduction

Proteasome inhibitor bortezomib (Btz) is approved by the FDA for the treatment of multiple myeloma and mantle cell lymphoma, and two newer proteasome inhibitors, carfilzomib (Cfz) and ixazomib, are approved for the treatment of multiple myeloma. Several trials have demonstrated the clinical efficacy of Btz for the treatment of acute lymphoblastic leukemia (ALL), albeit in combination with a standard chemotherapy^1–3^, and Cfz is undergoing clinical trials for the treatment of ALL^4^.

More than one-third of infant ALL is driven by the t(4;11) translocation, which leads to the expression of the mixed lineage leukemia 1 (MLL)–AF4 fusion protein. Although patients diagnosed with this type of leukemia respond well to initial chemotherapy treatment, they often relapse, and the five-year survival rate is about 50%^5^. MLL–AF4 expressing cells isolated from relapsed and refractory patients are highly sensitive to pharmacologically relevant concentrations of Btz ex vivo in the presence of bone marrow stromal cells^6^, and Btz was effective against patient-derived xenografts (PDXs) of MLL–AF4 leukemias^7^. One young adult patient achieved complete remission after single-agent treatment with Btz; however, the treatment was discontinued due to toxicities^8^.

**Figure 1:**
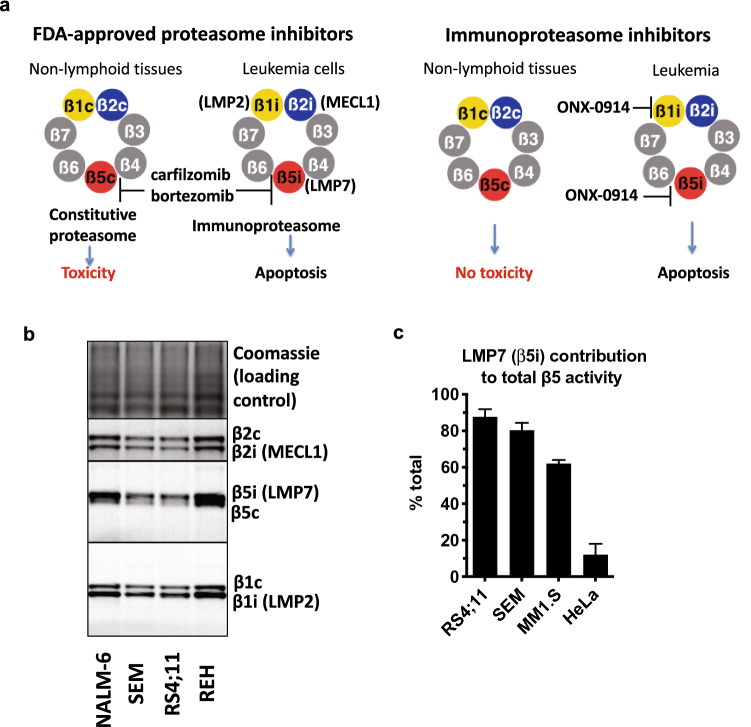
ALL cell lines express high levels of immunoproteasomes. (**a**) The principle of replacing FDA-approved proteasome inhibitors with immunoproteasome inhibitors for the treatment of ALL. Catalytic β-subunits of the proteasome are colored, non-catalytic β-subunits are gray. (**b**) Active proteasome subunits were labeled in cell extracts with proteasome-specific activity-based probes, resolved on SDS-PAGE, and detected by fluorescent imaging. Uncropped images of the gel, which also contain a second biological replicate, are presented in Fig. S1. The β5i/β5c image was stretched vertically to improve resolution between subunits. Subunit assignments are based on a pattern in HeLa cells (no immunoproteasomes) and inducibility of immunoproteasomes by interferon γ, which are shown in Fig. S2a, together with an additional biological replicate for SEM and RS4;11 cells. (**c**) To determine the contribution of LMP7 to the total activity of proteasome ß5 sites, cell extracts were treated with a highly specific LMP7 inhibitor LU-015i (5 μM, Fig. S2b) and cleavage of Suc-LLVY-amc was measured.

Proteasomes are large multi-subunit proteolytic complexes that play a crucial role in cellular protein homeostasis^9^. MLL–AF4 is an oncogenic short-lived protein but is pro-apoptotic when overexpressed. It is rapidly turned over by the proteasome, and treatment with Btz increases its expression to pro-apoptotic levels^8^. ALL cells predominantly express immunoproteasomes^10,11^_._. Immunoproteasomes are different from the ubiquitously expressed constitutive proteasomes, which predominate in the majority of non-lymphoid tissues. Both types of proteasomes share non-catalytic subunits but have three distinct pairs of active sites (Fig. 1a). The constitutive proteasome contains the ß5c (PSMB5), ß1c (PSMB6), and ß2c (PSMB7) catalytic subunits, which are replaced in the immunoproteasomes with LMP7 (ß5i, PSMB8), LMP2 (ß1i, PSMB9), and MECL1 (ß2i, PSMB10) subunits^12^. In addition, the so-called hybrid proteasomes contain two LMP7 subunits, two ß2c subunits, and either two LMP2 subunits or two ß1c subunits^13^. LMP7 and ß5c are the most important subunits for protein breakdown and the primary targets of the FDA-approved proteasome inhibitors, which inhibit them with similar potency^9^.

Since FDA-approved proteasome inhibitors inhibit the constitutive proteasomes, their clinical effectiveness is limited by toxicities, which is of particular concern for pediatric use. Although peripheral neuropathy, the most common side effect of Btz, is most likely an off-target effect^14^, inhibition of constitutive proteasomes is the most likely cause of gastrointestinal, renal, and cardiac toxicities^15–18^. Cardiac toxicity is a major concern for pediatric patients because it may generate long-term cardiac complications. Btz causes long-term testicular damage and permanently impairs bone growth in young mice^19,20^, raising the possibility of additional pediatric-specific toxicities. The tissues associated with on-target toxicities predominantly express constitutive proteasomes^13,21^, and selective inhibition of immunoproteasomes should reduce or eliminate much of these toxicities (Fig. 1a). Mice lacking catalytic subunits of immunoproteasomes are viable^22^, while deletion of constitutive subunits is lethal^23^. ONX-0914, an inhibitor of the LMP7 and LMP2 subunits of immunoproteasomes, has activity in multiple models of autoimmune diseases in mice^24,25^, and we found that it induces apoptosis in myeloma cells at pharmacologically relevant concentrations^26^. Other immunoproteasome inhibitors have also demonstrated activity in MM^27,28^. KZ-616, a derivative of ONX-0914^29^, is undergoing phase 2 clinical trials for the treatment of lupus. Although normal platelets and lymphocytes express immunoproteasomes^30,31^, hematologic toxicities (e.g., thrombocytopenia and neutropenia) were not observed in the phase I trial of KZ-616, suggesting the existence of a therapeutic window. Multiple assays to analyze the relative expression of constitutive and immunoproteasomes in patient samples are available^10,11,31^. They can be easily adopted for the selection of patients whose tumors predominantly express immunoproteasomes.

This paper demonstrates the efficacy of immunoproteasome inhibitor ONX-0914 in B-ALL cells bearing the MLL–AF4 translocations in vivo and in vitro, and in T-ALL cells.

## Results

### ALL cell lines express high levels of immunoproteasomes

Prior work of the Cloos laboratory used westerns to analyze the LMP7:ß5c ratio in primary ALL cells from ~ 50 patients. The ratio exceeded 10:1 in the majority of samples and even approached 100:1 in some patients^10,11^, making them excellent candidates for treatment with immunoproteasome inhibitors. In an independent study, the Driessen and Overkleeft laboratories have obtained similar results using activity-based probes^10,11^. To determine the LMP7:ß5c, LMP2:β1c, and MECL1:β2c expression ratios, we treated cell extracts from four cell lines, RS4;11, SEM, REH, and NALM-6 with activity-based probes^10,11^, and resolved labeled subunits on an SDS-PAGE. As can be seen in Fig. 1b, the LMP7 band was clearly detectable in all cells, and the PSMB5 (ß5c) band had much weaker intensity. Active LMP2 (ß1i) was also expressed at a higher level than its constitutive counterpart β1c (PSMB6). MECL1 (ß2i) and ß2c (PSMB6) subunits were expressed at approximately equal ratios, suggesting that these cells also express hybrid proteasomes, which contain LMP7 (ß5i), PSMB7 (ß2c), and either PSMB6 (ß1c) or LMP2 (ß1i) subunits^13^. To corroborate these findings, we studied the effect of a highly specific inhibitor of the LMP7 (ß5i) subunit of immunoproteasomes, LU-015i^32^, on the cleavage of the commonly used peptide substrate, Suc-LLVY-amc, which is cleaved by ß5c and LMP7 at approximately equal rates. This analysis was limited to RS4;11 and SEM cells, two lines expressing MLL–AF4 fusion protein, the primary focus of this study. LU-015i, a more specific inhibitor of LMP7 than ONX-0914, was used at a concentration that completely blocked LMP7 activity but did not inhibit PSMB5 (Fig. S2b). LU-015i treatment blocked 90% of total ß5 activity in RS4;11 cells and 75% in SEM cells, indicating that most proteasomes in these cells contain the LMP7 subunit (Fig. 1c). LMP7:PSMB5 ratio in these lines was higher than in MM1.S cells (Fig. S2a), which we previously used to demonstrate ONX-0914 activity in multiple myeloma^26^. Thus, the relative expression of LMP7 and ß5c in RS4;11 and SEM cells are similar to the ratio in patient cells known from the literature^10,11^.

### Pharmacologically relevant concentrations of ONX-0914 induce apoptosis in MLL–AF4 cells

**Figure 2:**
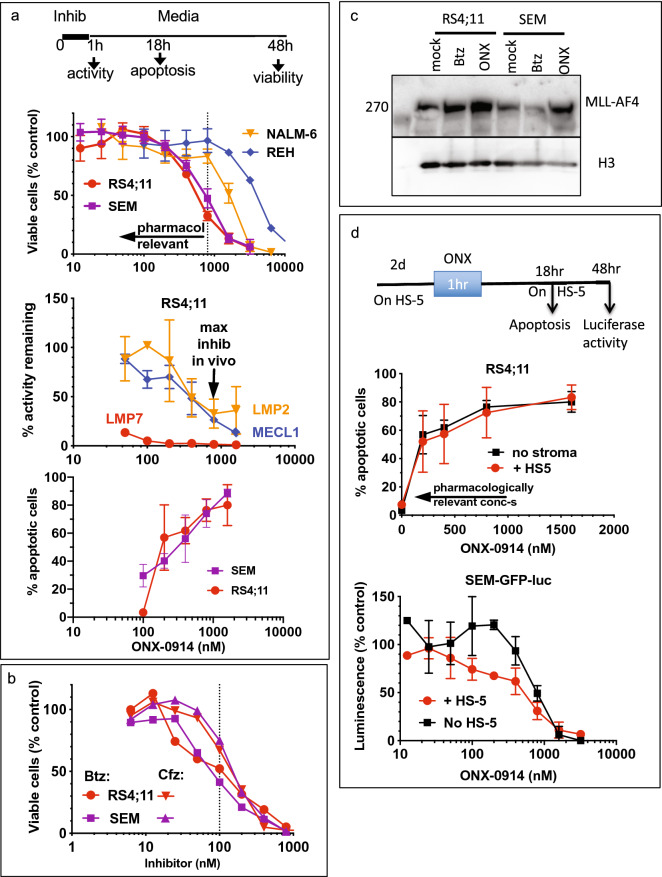
MLL–AF4 cell lines are sensitive to pharmacologically relevant concentrations of ONX-0914. (**a**) Cells were treated with ONX-0914 for 1 h and cultured for 2 days before assessing cell viability with Alamar Blue (top panel, n = 7, SEM and RS4;11; n = 2, other), or cultured for 17 h before measuring apoptosis (bottom panel, n = 2, SEM; n = 3, RS4;11). Alternatively, cells were harvested for activity measurements immediately after 1 h treatment (middle panel, n = 2), LMP7 and LMP2 activity was measured with fluorogenic substrates, MECL1 inhibition was determined using BODIPY(TMR)-NC-005 probe. Arrows and dashed lines indicate pharmacologically relevant concentrations. (**b**) Cells were treated with Btz or Cfz for 1 h and cultured for 2 days before assessing cell viability with Alamar Blue (n = 2). (**c**) Cells were treated with 10 nM Btz or 800 nM ONX-0914 for 4 h. MLL–AF4 expression was analyzed by western using histone H3 as a loading control. Uncropped images of the blot are presented on Fig. S3. (**d**) RS4;11 or SEM-GFP-luc cells were cultured with HS-5 stromal cells for 48 h, treated with ONX-0914 for 1 h in the absence of HS-5, and then cultured with HS-5 cells for the times indicated. Apoptosis of RS4;11 cells was measured by flow-cytometry (top panel, n = 3). Size-based gating was used to distinguish between RS4;11 and HS-5 cells (see Fig. S4). Luciferase assay was performed to access viability of luciferase-expressing SEM-GFP-luc cells (n = 2). Bottom panel in (**a**) and top panel in (**d**) contain the same data for RS4;11 cells treated in the absence of stroma.

We found that brief exposure to ONX-0914 caused a concentration-dependent loss of viability of ALL cells (Fig. 2a, top panel). In these experiments, we pulse-treated cells with ONX-0914 for 1 h, removed the drug, and measured viability 48 h later. The 1 h pulse incubation better mimics the clinical scenario than the continuous exposure usually used for in vitro experiments, because the concentration of inhibitors in the patients’ blood peaks within an hour after the bolus treatment and then declines rapidly^33^. There were notable differences in sensitivity to ONX-0914 between cell lines. The two cell lines that express the MLL–AF4 fusion protein and are highly sensitive to Btz^8^, RS4;11 and SEM, were the most sensitive. REH cells, previously reported to be substantially less sensitive to Btz than SEM and RS4;11 cells^8^, were also found to be less sensitive to ONX-0914 (Fig. 2a), despite expressing a similar LMP7:PSMB5 ratio (Fig. 1b). Finally, NALM-6 cells, which were reported to be as sensitive to Cfz as RS4;11 cells^34^, were less sensitive to ONX-0914 (Fig. 2a). Thus, cells expressing MLL–AF4 fusion proteins are more sensitive to the immunoproteasome inhibitor than ALL cells that do not express this fusion.

At maximally tolerated doses in mice, ONX-0914 inhibits > 95% of LMP7 and 60–80% of LMP2 activity^24^. To determine which concentrations of ONX-0914 cause this pharmacologically relevant inhibition, we measured the inhibition of individual active sites immediately after one-hour incubation of RS4;11 cells with ONX-0914 and found that ~ 0.8 μM ONX-0914 causes near-complete inhibition of ß5i (LMP7) sites and 60–80% inhibition of ß1i (LMP2) sites (Fig. 2a, middle panel). Therefore, we considered concentrations below 0.8 μM pharmacologically relevant. These concentrations reduced viability (Fig. 2a, upper panel) to a similar extent as 100 nM concentrations of Btz and Cfz (Fig. 2b), which we have previously shown to cause in vivo achievable inhibition of the proteasome^33,35^. We confirmed that incubation with pharmacologically relevant concentrations of ONX-0914 induced apoptosis of both cell lines (Fig. 2a, bottom panel). We also found that incubation with ONX-0914 and Btz caused a similar up-regulation of the MLL–AF4 fusion protein (Fig. 2c). This protein is pro-apoptotic when overexpressed, and its’ accumulation is responsible for the exquisite sensitivity of these cells to Btz^8^. Thus, ALL cells that express the MLL–AF4 fusion protein are sensitive to pharmacologically relevant concentrations of ONX-0914.

To determine whether the presence of stroma affects sensitivity of ALL cells to ONX-0914, we cultured RS4;11 and luciferase-expressing SEM cells with HS-5 stromal cells, treated them with ONX-0914 for 1 h, and re-plated them on stromal cells. We then determined apoptosis of RS4;11 cells by flow-cytometry using caspase-3 probe and measured luciferase activity in the surviving SEM cells (Fig. 2d). In both cases, stroma did not affect sensitivity to ONX-0914. Thus, stromal cells do not protect ALL cells from ONX-0914 induced apoptosis.

### Co-inhibition of ß2 sites enhances the anti-neoplastic activity of ONX-0914

**Figure 3:**
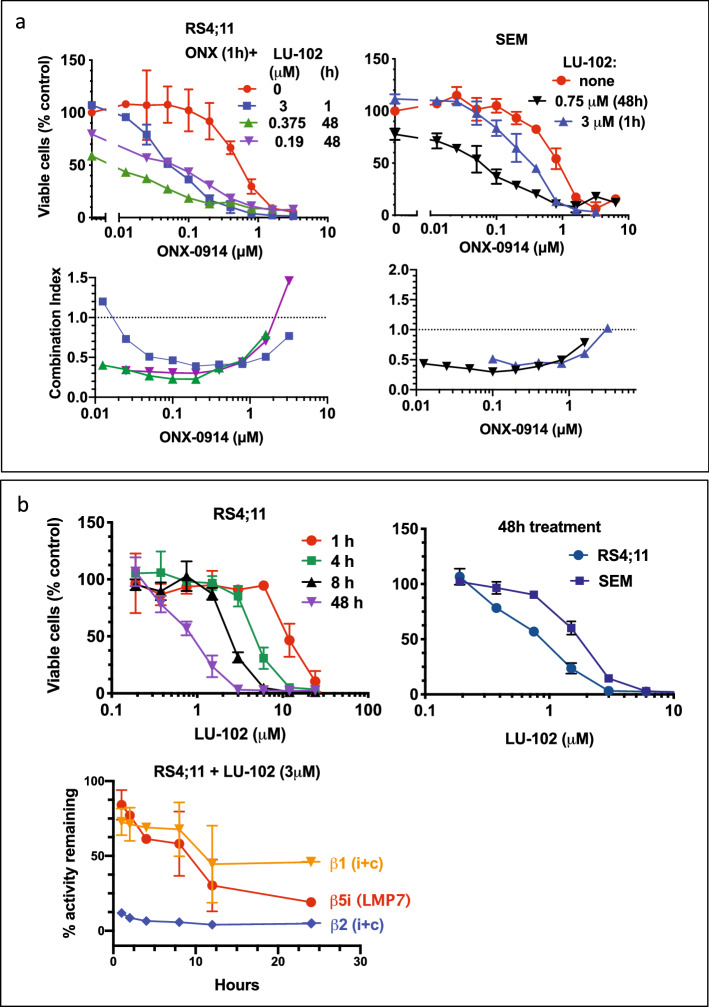
ß2 sites are co-targets for the treatment of ALL. (**a**) Cells were co-treated with ONX-0914 and LU-102 for 1 h, or treated with ONX-0914 for 1 h, followed by 47 h treatment with LU-102, and cell viability was assessed with Alamar Blue (top panels, n = 2–5). Combination indexes were calculated using CalcuSyn software (bottom panels). (**b**) Top panels, cells were treated with LU-102 for times indicated (left panel) or for 48 h (right panel). Cell viability was assessed with Alamar Blue 48 h after start of the treatments (n = 2). Bottom panel, inhibition of different active sites was measured with fluorogenic substrates in extracts of cells continuously treated with LU-102 (n = 2).

We found previously that ß1 and ß2 sites’ inhibitors sensitize cells to PSMB5 (ß5c) and LMP7 inhibitors^10,26,35–38^. Similar to these earlier observations, we found that LU-102, an inhibitor of MECL1 and ß2c^39^, synergizes with ONX-0914 (Fig. 3a). The most robust synergy was observed at pharmacologically relevant concentrations, including 100 and 200 nM concentrations of ONX-0914, which specifically inhibited LMP7 sites (compare Fig. 3a with the middle panel on Fig. 2a). We also noted that RS4;11 cells were sensitive to a single-agent treatment with LU-102 (Fig. 3b), although maximal activity was observed when LMP7 sites were co-inhibited. Thus, the ß2 sites (MECL1 and ß2c) are also targets for the treatment of ALL.

### Increasing duration of treatment increases cytotoxicity

**Figure 4:**
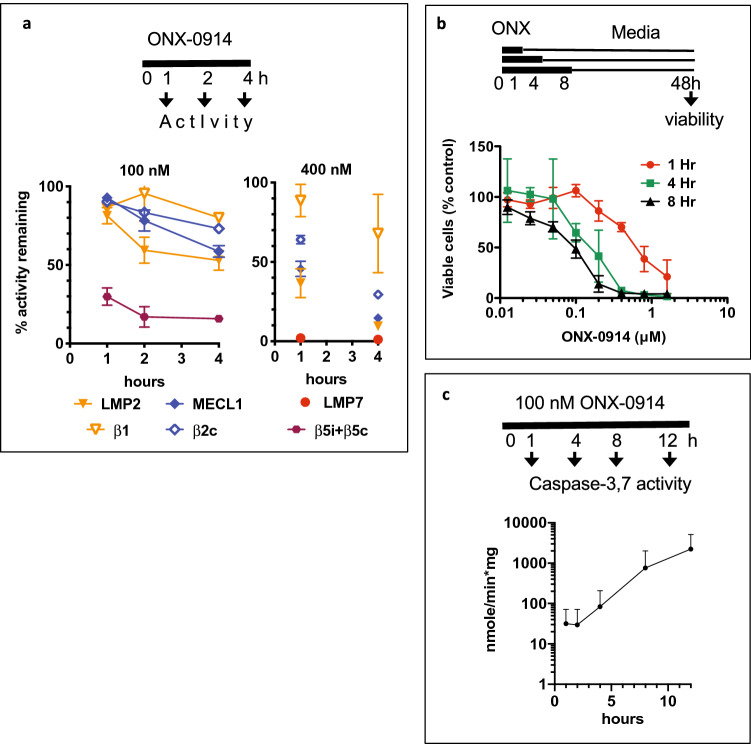
Longer treatment increased proteasome inhibition and cytotoxicity. (**a**) RS4;11 cells were continuously treated with indicated concentrations of ONX-0914. At times indicated inhibition of active sites was determined with either substrates (combined ß5 activity on the left panel, LMP7 and LMP2 activity on the right panel) or a mixture of BODIPY(FL)-NC-001 and BODIPY(TMR)-NC-005 activity-based probes (all others, n = 2). Suc-LLVY-amc was used to measure total ß5 activity on the left panel, the residual activity at 4 h is due to ß5c. (**b**) RS4;11 cells were treated for times indicated, followed by culture in drug-free media. 48 h after the start of the treatment viability was measured with Alamar Blue (top panel, n = 2). (**c**) Caspase-3,7 activity was measured in extracts of RS4;11 cells continuously treated with 100 nM ONX-0914 (n = 3).

Another way to increase the inhibition of LMP2 and ß2 sites is to increase exposure to the inhibitor. Indeed, longer incubation with ONX-0914 increased inhibition of LMP2 and MECL1 (Fig. 4a). Consistent with increased inhibition, cell viability decreased in cells exposed to ONX-0194 for 4 h or 8 h compared to 1 h exposure (Fig. 4b), and 4 h exposure to 400 nM ONX-0914 resulted in a complete loss of viability. The difference between 4 and 8 h exposure was not significant, perhaps because apoptosis is already occurring 4 h after incubation start (Fig. 4c). Thus, the development of a formulation that would allow for a longer exposure to the drug should increase the anti-leukemia properties of immunoproteasome inhibitors.

### ONX-0914 is active in vivo

**Figure 5:**
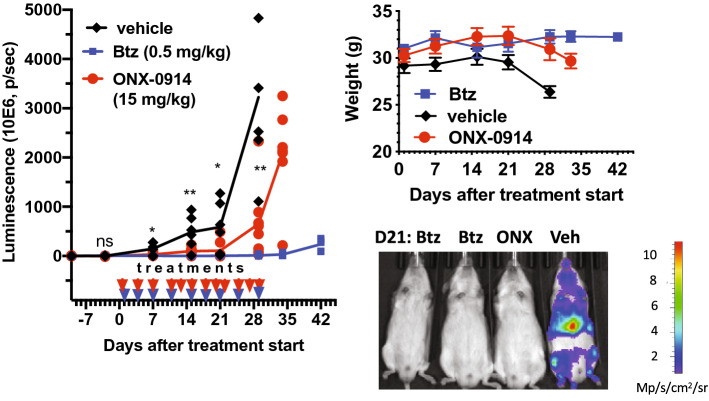
ONX-0914 is active in vivo. NSG mice were injected with SEM-GFP-luc cells 10 days prior to the start of subcutaneous treatments with Btz (n = 8), ONX-0914 (n = 8), or vehicle (10 mM sodium citrate, pH 6.0, 10% Captizol; n = 6). The sample size was based on past experience. The control group originally contained 7 animals, but one animal died of an unrelated cause and was not included in the analysis. ONX-0914 and vehicle-treated groups were compared by unpaired t-test; *0.01 < p < 0.05; **p < 0.01. Lines on the left panel connect mean values. A representative image of the animals on d21 is shown at the bottom panel. All other images are presented on Fig. S5.

To test the efficacy of ONX-0914 in vivo, we intravenously injected male NSG mice with luciferase-expressing SEM cells, resulting in the formation of tumors in the spleen and bone marrow (Figs. 5, S4). Once tumors were detected, we treated animals with Btz or ONX-0914. Btz (0.5 mg/kg) was used at the murine equivalent of human MTD, and ONX-0914 was used at 15 mg/kg, which is within the range of doses (6–20 mg/kg) used in murine models of autoimmune disease^24,25,40^. ONX-0914 significantly delayed tumor growth and was comparable to Btz during the initial 3 weeks of treatment. However, ONX-0914 treated tumors progressed faster than Btz-treated tumors. It is also worth noting that these doses of ONX-0914 and Btz did not show any efficacy in orthotopic xenografts of MM1.S myeloma cells^26^. Thus, inhibition of immunoproteasomes reduces MLL–AF4 leukemia proliferation in mice.

### MLL–AF4 cells recover immunoproteasome activity after treatment with sub-toxic concentrations of ONX-0914

**Figure 6:**
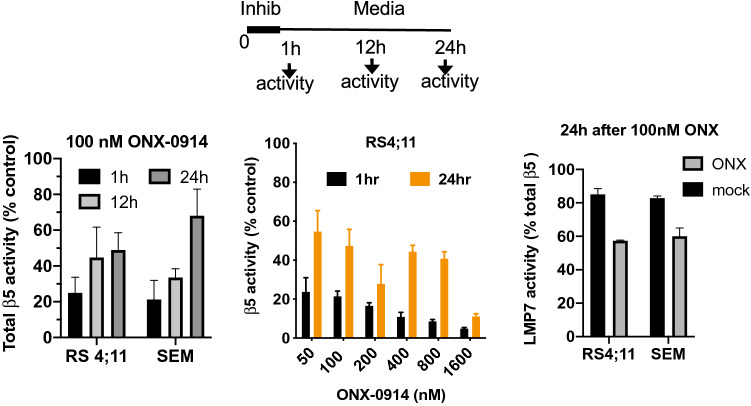
Immunoproteasome activity recovers after pulse treatment with sub-toxic doses of ONX-0914. The combined activity of LMP7 and ß5c sites was measured with Suc-LLVY-amc in extracts of ONX-0914-treated cells harvested immediately after 1 h treatment or after subsequent culturing in drug-free media (n = 3–7). On the right panel, extracts were treated with the highly specific immunoproteasome inhibitor LU-015i immediately before activity measurements (n = 2–3).

Cells treated with FDA-approved proteasome inhibitors are known to up-regulate the expression of constitutive but not immunoproteasomes^41–43^. PBMC and myeloma cells up-regulate constitutive proteasomes even when treated with immunoproteasome inhibitors^26,44^. Replacement of immunoproteasomes with constitutive proteasomes could potentially explain the earlier relapse of ONX-0914-treated tumors. We found that the total ß5 (PSMB5:LMP7) activity indeed recovers in cells pulse-treated with a sub-toxic concentration of ONX-0914 (100 nM) for 1 h. Mild 10–20% recovery was observed at 12 h after the treatment, and 60% of activity recovered at 24 h (Fig. 6, left panel). Recovery occured in cells treated with a variety of concentrations of ONX-0914, and only cells incubated with concentrations that cause a dramatic drop in viability and activity did not recover (middle panel). To determine whether LMP7 or PSMB5 are responsible for the recovery, we used LMP7-specific inhibitor LU-015i, similar to the experiment in Fig. 1c. We found that although LMP7 contribution to total ß5 activity was lower than in mock-treated cells, it was still responsible for 60% of recovered ß5 activity in ONX-0914-treated cells (Fig. 6, right panel). Thus, unlike PBMC and myeloma, ALL cells expressing the MLL–AF4 protein are capable of recovering immunoproteasome activity after incubation with immunoproteasome inhibitors.

## T-ALL cells are sensitive to immunoproteasomes inhibitors

**Figure 7:**
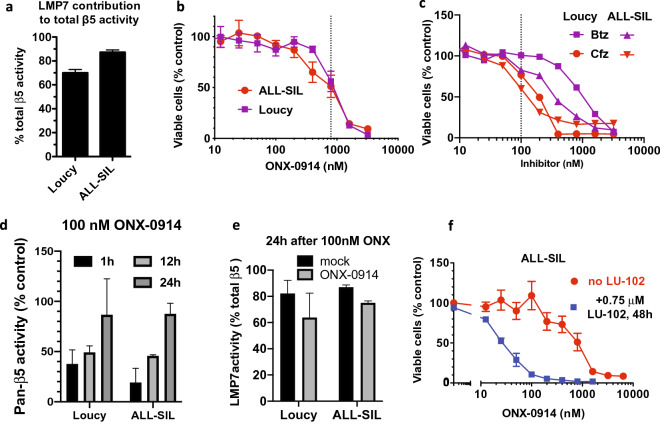
T-ALL cells are sensitive to pharmacologically relevant concentrations of ONX-0914. (**a**) LMP7 contribution to the cleavage of Suc-LLVY-amc was determined using LU-015i. (**b**,**c**) Cells were treated with ONX-0194 (n = 3–4), Btz, or Cfz (n = 2–3) for 1 h, and then cultured in the absence of inhibitors for 48 h, followed by an Alamar Blue assay. Dashed lines indicate concentrations that cause in vivo achievable levels of proteasome inhibition. (**d**,**e**) Recovery of a total ß5 (LMP7 + PSMB5) activity after 1 h treatment and LMP7 contribution to the recovery was determined as on Fig. 5 (n = 2–3). (**f**) ALL-SIL cells were treated with ONX-0914 for one hour, followed by 48 h treatment with 0.75 μM LU-102, and viability was measured with Alamar Blue (n = 2–3).

To explore the possibility that other subtypes of ALL may also be sensitive to ONX-0914, we have focused on T-ALL. Several studies have shown that patient-derived T-ALL cells are almost as sensitive to Btz as MLL–AF4 cells, and more sensitive than cells derived from subtypes of B-ALL that do not bear MLL–AF4 fusion^6,7^. Two T-ALL cell lines, LOUCY and ALL-SIL, expressed high ratios of LMP7 to PSMB5 (Fig. 7a) and were more sensitive to pharmacologically relevant concentrations of ONX-0914 than of Btz and Cfz (Fig. 7b,c). Similar to MLL–AF4 expressing B-ALL cells, T-ALL cells were capable of recovering immunoproteasome activity after treatment with sub-toxic concentrations (Fig. 7d,e). Cells were sensitized to ONX-0914 by sub-toxic concentrations of ß2 inhibitor LU-102 (Fig. 7f). Thus, the immunoproteasome is also a target in T-ALL.

## Discussion

Targeting immunoproteasomes is an experimental strategy for the treatment of autoimmune diseases and multiple myeloma. Here we provide the rationale for targeting immunoproteasomes in B-ALL cells expressing the MLL–AF4 fusion protein. Our data also suggests that immunoproteasome inhibitors will be effective against T-ALL, and perhaps other subtypes of B-ALL. Primary ALL cells are highly sensitive to proteasome inhibitors. The ratio of immuno- to constitutive proteasome in ALL is the highest among hematologic malignancies^10^. It can be easily tested in every patient by using activity-based probes as shown in this study or by ELISA-based assay developed by Proteolix^31^. As such, ALL may be the best candidate for clinical development of immunoproteasome inhibitors for the treatment of hematologic malignancies. In fact, we found that Btz and ONX-0914 are active in ALL xenografts at doses (Fig. 5) that were inactive in a similar orthotopic model of multiple myeloma^26^. Although ONX-0914 was not as effective as Btz in an animal model, it has to be kept in mind that this compound is a research tool that is not being developed clinically. While the t(4;11)(q21;q23) translocation that generates MLL–AF4 fusion protein can be used as a predictive marker of response, further work is needed to verify additional markers, such as deletion of IKZF1 or biallelic deletion of CDKN2A that were shown to sensitize pre-B-ALL to Btz and Cfz^34^.

This study also provides insight into the properties of the immunoproteasome inhibitors that are important for their anti-neoplastic activity. We confirm earlier findings in other immunoproteasome expressing tumor types that co-inhibition of either ß1 or ß2 sites dramatically improves cytotoxicity of LMP7 inhibitors. The major purpose of replacing FDA-approved proteasome inhibitors with immunoproteasome inhibitors is to reduce the toxicity associated with inhibition of constitutive proteasomes. Therefore, the ability to co-inhibit LMP7, LMP2, and MECL1 sites must be achieved without inhibiting the PSMB5 site of constitutive proteasomes. In this regard, ONX-0914 is a sub-optimal compound because it inhibits PSMB5 and LMP2 at similar concentrations^24,26^. We have discovered such selective pan-immunoproteasome inhibitors^32^; however, their pharmacologic properties must be improved. High specificity for the immunoproteasome should allow for more frequent administration than the twice weekly FDA-approved dosing for Cfz and Btz, once weekly for KZ-616, and thrice-weekly as we used in this study for ONX-0914. More frequent dosing should lead to longer suppression of activity and reduce the likelihood of recovery of activity. M3258, a novel highly specific inhibitor of LMP7 may satisfy these requirements because it is dosed daily and is capable of continuous suppression of activity in mice^45^. It is undergoing clinical trials for the treatment of multiple myeloma. We are currently testing this inhibitor in cell lines expressing the MLL–AF4 fusion protein, which will be followed by testing M3258 efficacy in patient-derived xenografts.

Previous data in the literature showing that PBMC and myeloma cells respond to the treatment of immunoproteasomes inhibitors by replacing immunoproteasomes with constitutive proteasomes raised concern that this can lead to the development of resistance^26,44^. Surprisingly, we found that immunoproteasomes are responsible for the bulk of activity recovered in ONX-treated cells expressing the MLL–AF4 fusion protein and in T-ALL cells. This unique ability of ALL cells to recover immunoproteasome activity can potentially spare normal immunoproteasome expressing cells from on-target toxicity and therefore increase the therapeutic window of immunoproteasome inhibitors in ALL.

To the best of our knowledge, this is the first study demonstrating activity of an immunoproteasome inhibitor in ALL expressing the MLL–AF4 fusion protein. We did not test ONX-0914 in patient samples or PDX and did not test it in combination with chemotherapy, because we consider it unnecessary to conduct such studies with a pre-clinical compound when two clinical stage agents, KZR-616 and M3258, of the class are available. To the best of our knowledge, the developers of these compounds are not yet planning to conduct clinical trials in ALL patients. We believe that this study provides a strong rationale for testing KZ-616 and M3258 in patient samples and PDX, and eventually in clinical trials of these immunoproteasome inhibitors for the treatment of ALL.

## Materials and methods

### Inhibitors and substrates

Carfilzomib and bortezomib were obtained from LC laboratories, ONX-0914 was obtained from MedChemExpress. LU-102, LU-015i, and activity-based probes were kindly provided by Drs. Bogdan Florea and Herman Overkleeft (Univ of Leiden, the Netherlands). Suc-LLVY-amc was obtained from Bachem. All other substrates were custom synthesized by China Peptide.

### Cell lines and cell culture experiments

RS4;11, LOUCY, HS-5, REH, NALM-6 and RPMI-8226 cells were obtained from the American Tissue Culture collection; SEM and ALL-SIL were obtained from DSMZ (Braunschweig, Germany). HS-5 cells were cultured in DMEM; all other cells were cultured in RPMI-1640 media. Both media were supplemented with 10% FBS, penicillin, streptomycin, and ciprofloxacin (0.2 μg/ml). Cell viability was assayed with resazurin (Alamar Blue, Sigma). Apoptosis was measured by flow-cytometry on BD Accuri C6 Plus flow-cytometer using CellEvent™ Caspase-3/7 Green Detection Reagent and SYTOX cell viability dye (Thermo). Data was analyzed using BD CSampler Plus software. Alternatively, caspase-3,7 activity was measured in extracts using Ac-DEVD-amc as described^36^. To measure luciferase activity, 1.6 mg/ml D-luciferin (GoldBio) was added to each well, and luminescence was measured after 5 min incubation at room temperature.

### Creation of SEM-GFP-luc cells

GFP and luciferase-expressing SEM cells (SEM-GFP-luc) were generated by transducing SEM cells with a Luciferase-T2A-eGFP lentiviral vector and subsequent sorting for GFP-positive cells. The lentiviral plasmid was cloned as previously described^46^. Briefly, the enhanced luc + gene was amplified from the pGL3 plasmid using primers engineered to harbor XbaI and BamHI sites at the 5′ and 3′ ends, respectively. This product was then ligated to a lentiviral transfer plasmid engineered to contain XbaI/BamHI in a polylinker, followed by a T2A-eGFP (containing a stop codon, eGFP cloned from pEGFP-N1) sequence. The plasmid was verified using Sanger sequencing. The full plasmid map is available in the supplementary files (Fig. S6).

### Proteasome activity measurements and identification

Frozen cell pellets were lysed in a buffer of 50 mM Tris–HCl, pH 7.5, 25% sucrose, 2 mM EDTA, 1 mM DTT, 1 mM ATP, 0.05% digitonin. Proteasome activity was measured in extracts of treated cells with Suc-LLVY-amc (ß5c and LMP7), Ac-ANW-amc (LMP7), Ac-WLA-amc (ß5c), Ac-nLPnLD-amc (ß1c and LMP2), Ac-APL-amc (LMP2) and Ac-RLR-amc (ß2c and MECL1) fluorogenic substrates^36^. It was normalized to the total protein content of the extract, which was determined using the Coomassie Plus—The Better Bradford Assay Reagent (Thermo). To distinguish between contribution of ß5c and LMP7 to the cleavage of Suc-LLVY-amc, extracts were preincubated with a highly LMP7-specific inhibitor LU-015i (5 μM) for 30 min at 37 °C immediately before activity measurements. Alternatively, occupancy of active sites was measured with activity-based probes as described^10,37^. In a three-probe-cocktail, ß1c (PSMB6) and LMP2 (ß1i) subunits are labeled with Cy5-NC-001, BODIPY(FL)-LU-112 labels MECL1 (ß2i) and ß2c (PSMB7), and LMP7 (ß5i) and ß5c (PSMB5) subunits were labeled with BODIPY(TMR)-NC-005-vs^10^. In a two-probe combination, LMP7, ß5c, MECL1 and ß2c subunits were labeled with BODIPY(TMR)-NC-005, and ß1c and LMP2 were labeled with BODIPY(FL)-NC-001^37,47^. Labeled subunits were resolved in 10% Bis–Tris SurePAGE™ (Genscript) and imaged on c600 gel imager (Azure Biosystems).

### Western blot analysis

Nuclear extracts were prepared using NE-PER™ nuclear extraction reagent (Thermo Fisher) separated on 4–12% Bis–Tris SurePAGE™ (Genscript) using MOPS running buffer, and transferred to a nitrocellulose membrane using methanol-free transfer buffer^48^. The MLL–AF4 fusion protein was revealed using D2M7U antibody to N-terminal antigen of MLL-1; histone H3 was detected with D2B12 antibody (Cell Signaling). We used HRP-conjugated secondary antibodies (Cell Signaling) and Super Signal West Femto Maximum Sensitivity substrate (Thermo Scientific) for band visualization.

### Equipment and setting

All gel and blot images were acquired on a c600 multi-mode imager (Azure Biosystems), using the “Autoexpose” feature, except for images in the blue channel that used 30 s exposure. Fluorescent bands were quantified using Image Studio Lite software (LiCOR). Input levels for all gel images in the figures were adjusted in Adobe Photoshop to ensure gray background. Cytation 5 multi-mode plate reader (BioTech) was used for fluorescent assays of the proteasome and caspase activity, Alamar Blue assay of cell viability, and for Bradford assays.

### Animal studies

6–8 week-old male NSG (NOD.Cg-*Prkdc*^*scid*^* Il2rg*^*tm1Wjl*^/SzJ, e.g. Nod/Scid/IL-2 receptor γ-chain knockout, Jackson Laboratory) mice were injected intravenously with 1 million SEM-GFP-luc cells. Animals were randomly assigned to treatment/vehicle groups. The sample size (7–8 animals/group) was based on past experience. Dosing and frequency are indicated on Fig. 5. We did not perform blinding because the primary readout of the experiment was an objective measurement of luminescence of luciferase-expressing tumors. Mice were injected intraperitoneally with D-Luciferin (150 mg/kg) once weekly and imaged on Xenogen IVIS-200 imaging system under isoflurane anesthesia. Photon counts of the whole animal were determined using Living Image software (Perkin Elmer). All animal procedures were carried out according to US Public Health Service Policy on Care and Use of Laboratory Animals and were approved by Dartmouth College IACUC (protocol #2203). The results of the study are reported according to ARRIVE guidelines.

### Data analysis

All values shown on the graphs (except the left panel on Fig. 5, which shows value for each animal in the group) indicate means ± S.E.M of several biological replicates, the exact number of which (n) is indicated in the caption. GraphPad Prism was used for graphing and analysis.

## Supplementary Information

Supplementary Information.
