# Supplementary material for: Activity of immunoproteasome inhibitor ONX-0914 in acute lymphoblastic leukemia expressing MLL–AF4 fusion protein
Source: Sci Rep. 2021 May 25;11:10883. doi: 10.1038/s41598-021-90451-9 (PMC8149845; doi:10.1038/s41598-021-90451-9)
Supplement: Supplementary file 1 — Supplementary Information. [file 41598_2021_90451_MOESM1_ESM.pdf]

Activity of immunoproteasome inhibitor ONX-0914 in acute lymphoblastic leukemia expressing MLL-AF4 fusion protein

Tyler W. Jenkins[1], Sondra L. Downey-Kopyscinski[2,3*], Jennifer L Fields[2,4], Gilbert J. Rahme[2,3*], William C. Colley[1*], Mark A. Israel[2,4*] Andrey V. Maksimenko[1], Steven N. Fiering[2,4], Alexei F. Kisselev[1]

[1]Department of Drug Discovery and Development, Harrison School of Pharmacy, Auburn University, Auburn AL; Norris Cotton Cancer Center[2] and Departments of Molecular and Systems Biology[3], and Microbiology and Immunology[4], Geisel School of Medicine, Dartmouth College, Lebanon, NH;

*Present affiliations: SLDK - Rancho Biosciences, San Diego, CA; GJR- Massachusetts General Hospital, Harvard Medical School, Boston, MA, and Broad Institute of Harvard and MIT, Cambridge, MA; WCC - ScribeAmerica, Huntsville Hospital, Huntsville, AL; MAI- Israel Cancer Research Fund, New York, NY

Running title: Immunoproteasome inhibitors for the treatment of ALL

Corresponding author: Alexei F. Kisselev, Pharmacy Research Building, 720 S. Donahue Dr., Auburn University AL 36849. Email: AFK0006@auburn.edu; Phone: 334-844-7356; fax 334-844-8331

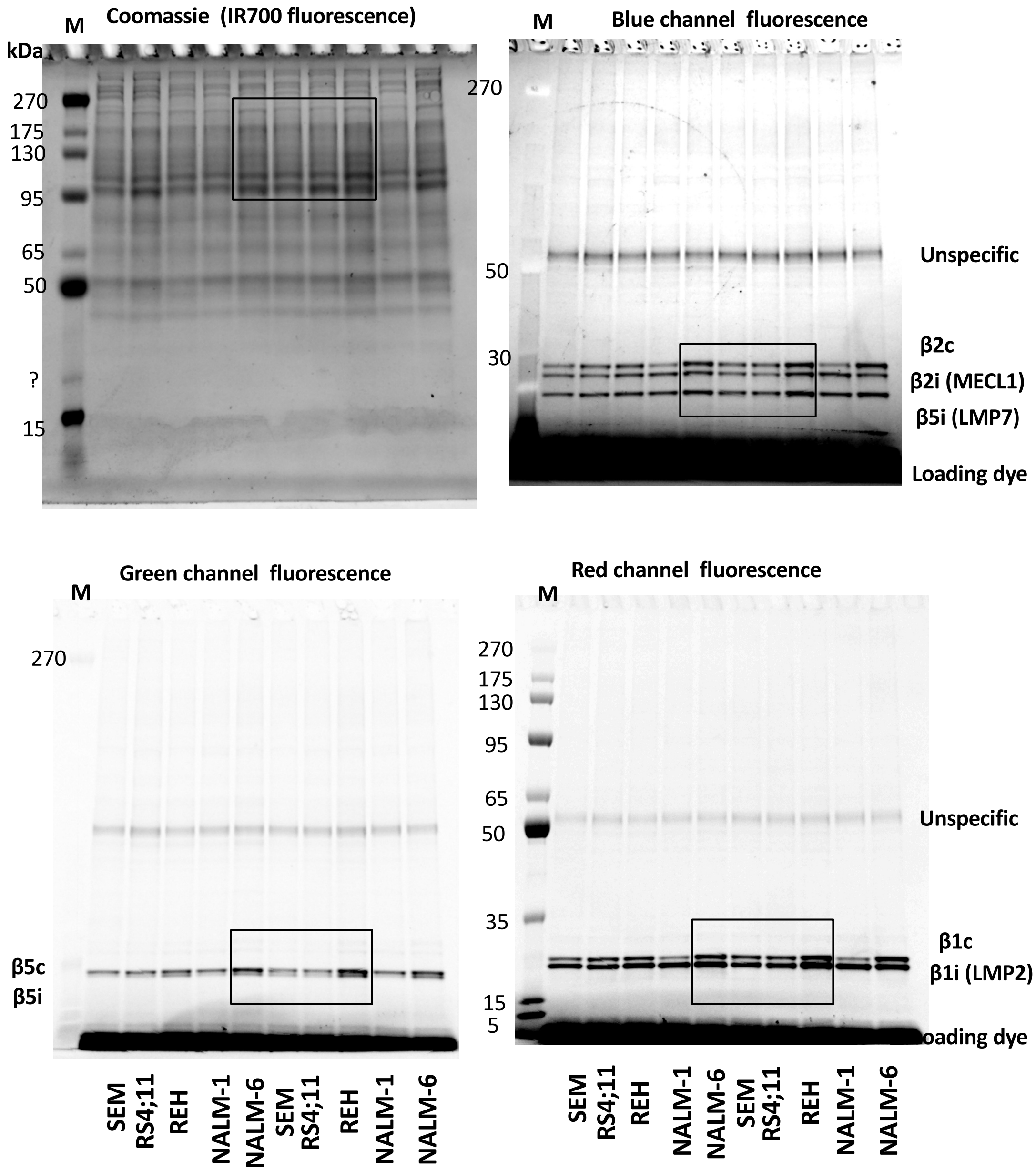

Figure S1**. Full images of the gel from Fig. 1b.** Croped are used in Fig. 1b are indicated with a box. Biological replicates are shown. The cropped are in the green channel image was expanded vertically on Fig. 1b to improve resolution between β5i and β5c subunits. NALM-1, a pediatric CML cell line in a lymphoid blast crisis, is included because we considered it an ALL cell line based on description on ATCC web site. The Coomassie staining was performed after RBG fluorescent imaging for probe-labeled subunits. Coomassie-stained band were detected by fluorescence in the IR700 channel. Input levels of the original images were adjusted in Photoshop so that edges of the gel are visible.

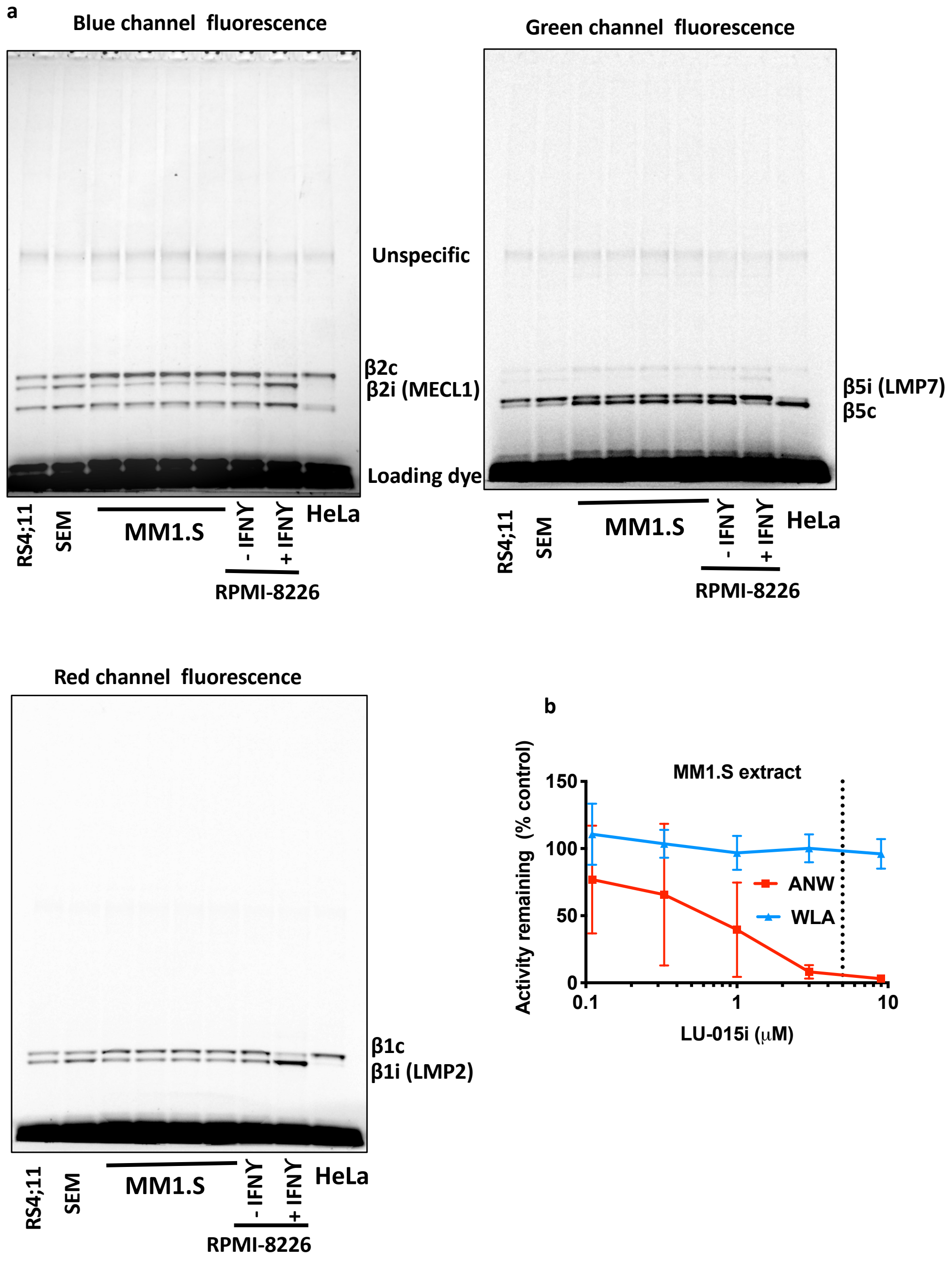

Figure S2. **Expression of immunoproteasomes in different cell lines. A**. Expression of different proteasome subunits was analyzed in cells extracts as in Fig. 1b. Extracts from RS4;11 and SEM cells are different samples than on Fig. 1b. MM1.S samples are biological replicates. RPMI-8226 cells were treated with IFNγ, which induces immunoproteasomes, for 4 days as described [26]. **B**. Extracts of MM1.S cells, which express approximately equal amounts of LMP7 and PSMB5 subunits, were treated with LU-015i for 30 minutes at 37°C (n=3). Dashed line indicate concentration used in assays in Figs. 1c, 6, 7a, 7e.

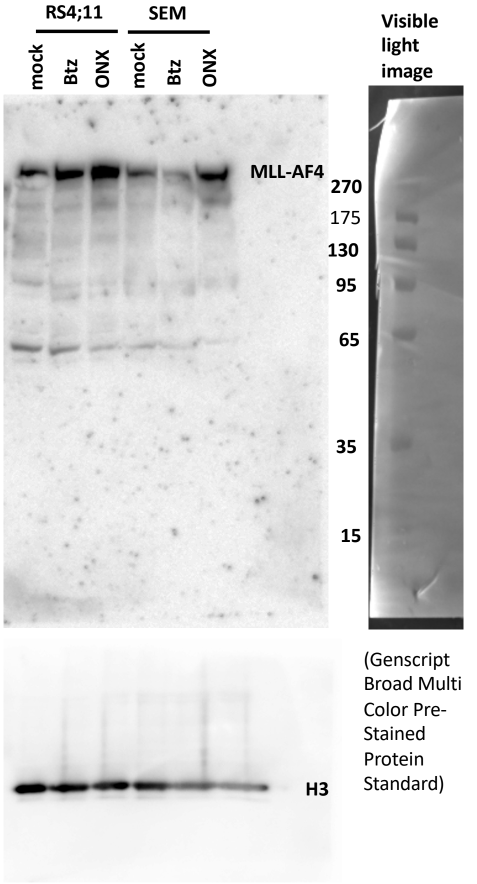

Figure S3. **Full images of the blot on Fig. 2c.** After probing the membrane with MLL-1 antibody, the bottom part of the membrane was cut off and re-probed with histone H3 antibody.

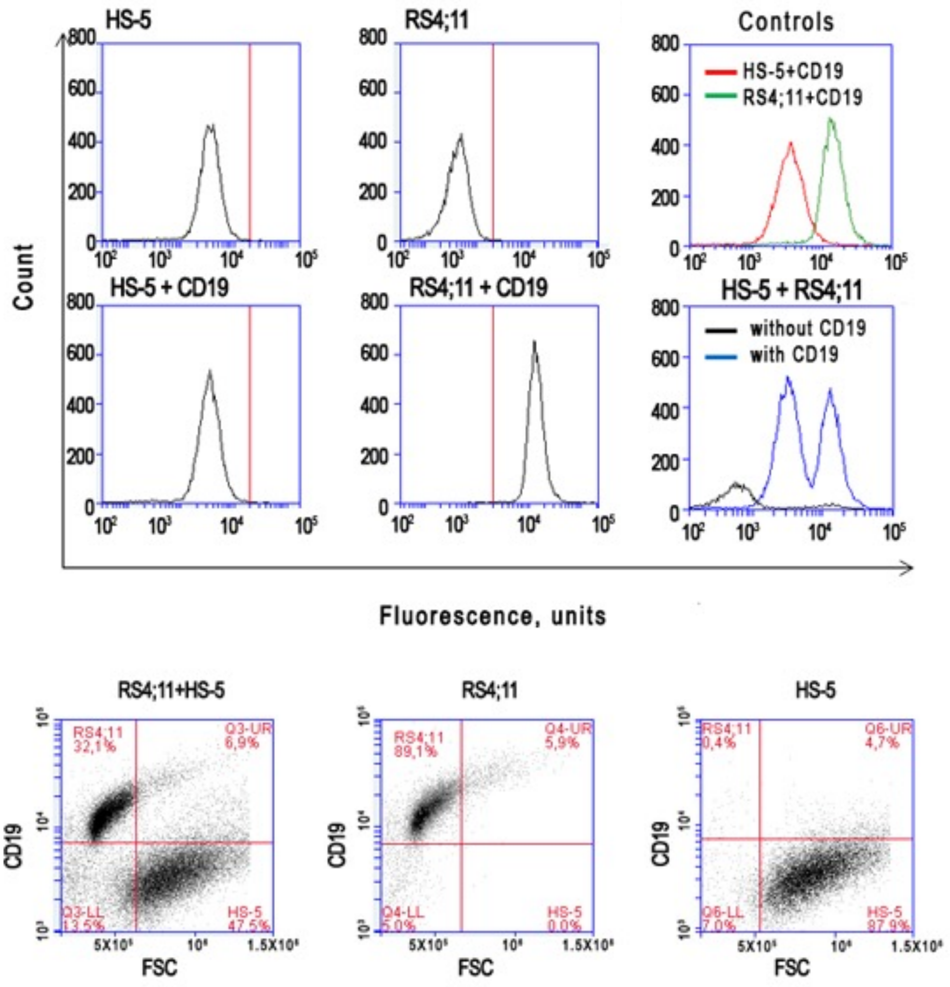

Figure S4. **Validation of cell-size based gating in flow-cytometry assays of apoptosis in the mixture of RS4;11 and HS-5 cells.** Cells were harvested and stained with PE-conjugated antibodies B-cell marker CD19 (Southern Biotech). Note that CD19-positive RS4;11 cells are smaller than CD19-negative HS-5 cells.

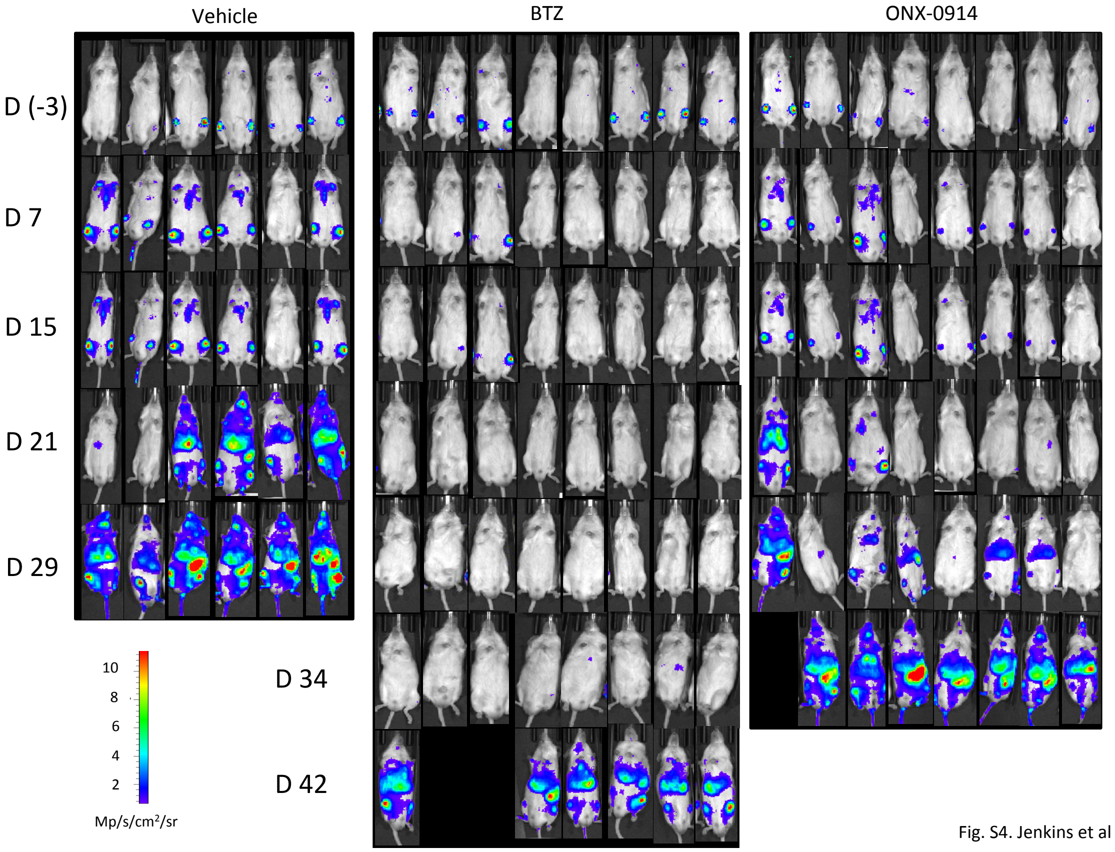

Figure S5. **ONX-0914 is active in vivo.** Individual images of animals from experiment on Fig. 5. 10s exposure.

Figure S6. The map and sequence of the Luciferase-T2A-eGFP lentiviral vector, that was used to create SEM-GFP-luc cells.

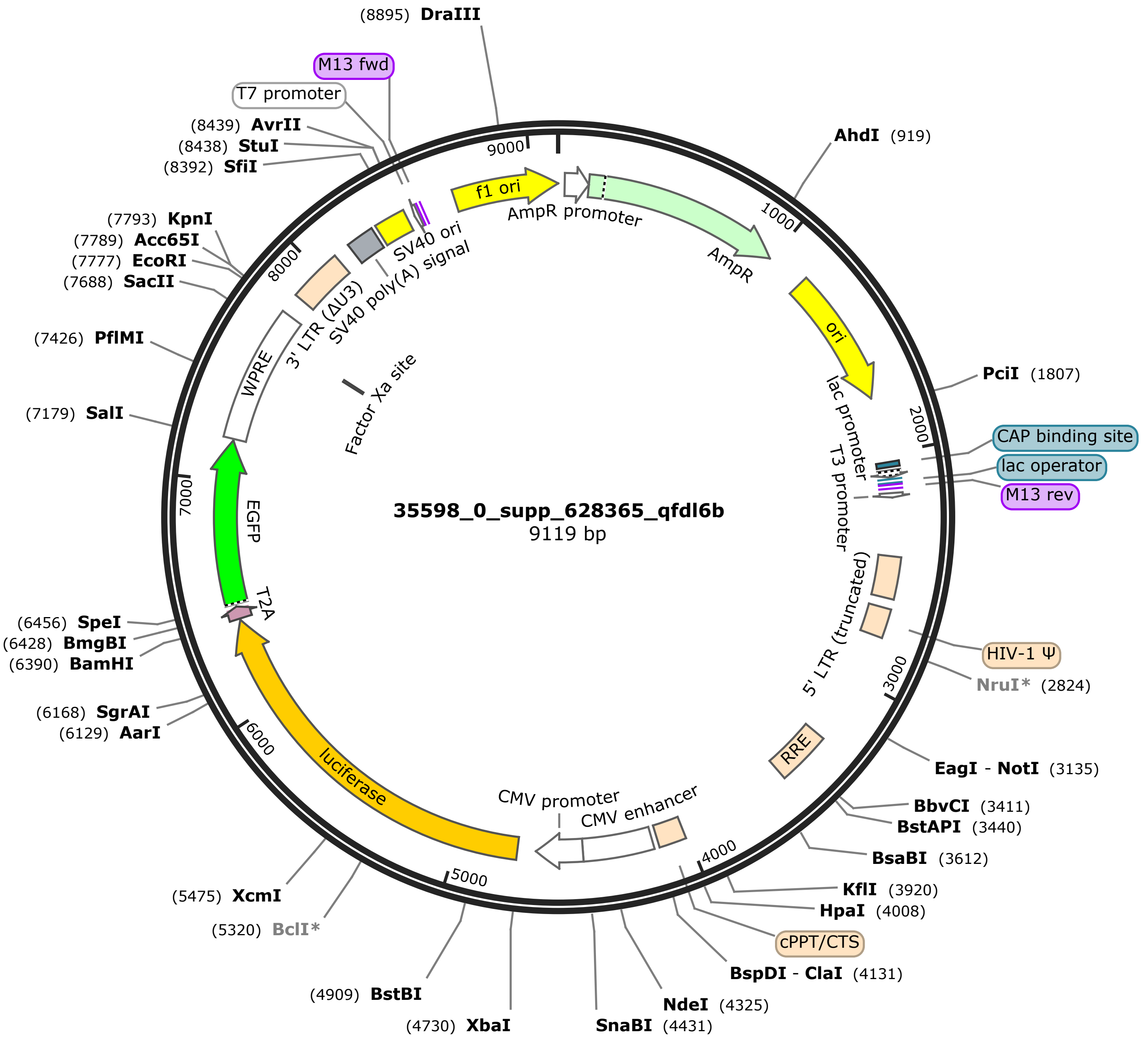

5′ caggtggcacttttcggggaaatgtgcgcggaacccctatttgtttatttttctaaatacattca 65
3′ gtccaccgtgaaaagcccctttacacgcgccttggggataaacaaataaaaagatttatgtaagt

AmpR promoter

aatatgtatccgctcatgagacaataaccctgataaatgcttcaataatattgaaaaaggaagag 130
ttatacataggcgagtactctgttattgggactatttacgaagttattataactttttccttctc

AmpR promoter

tatgagtattcaacatttccgtgtcgcccttattcccttttttgcggcattttgccttcctgttt 195
atactcataagttgtaaaggcacagcgggaataagggaaaaaacgccgtaaaacggaaggacaaa

1 Met Ser Ile Gln 5 His Phe Arg Val Ala 10 Leu Ile Pro Phe Phe 15 Ala Ala Phe Cys Leu 20 Pro Val

signal sequence

AmpR

AmpR promoter

ttgctcacccagaaacgctggtgaaagtaaaagatgctgaagatcagttgggtgcacgagtgggt 260
aacgagtgggtctttgcgaccactttcattttctacgacttctagtcaacccacgtgctcaccca

Phe Ala His 25 Pro Glu Thr Leu Val 30 Lys Val Lys Asp Ala 35 Glu Asp Gln Leu Gly 40 Ala Arg Val Gly

signa...

AmpR

tacatcgaactggatctcaacagcggtaagatccttgagagttttcgccccgaagaacgttttcc 325
atgtagcttgacctagagttgtcgccattctaggaactctcaaaagcggggcttcttgcaaaagg

Tyr 45 Ile Glu Leu Asp Leu 50 Asn Ser Gly Lys Ile 55 Leu Glu Ser Phe Arg 60 Pro Glu Glu Arg Phe 65 Pro

AmpR

aatgatgagcacttttaaagttctgctatgtggcgcggtattatcccgtattgacgccgggcaag 390
ttactactcgtgaaaatttcaagacgatacaccgcgccataatagggcataactgcggcccgttc

Met Met Ser Thr 70 Phe Lys Val Leu Leu 75 Cys Gly Ala Val Leu 80 Ser Arg Ile Asp Ala 85 Gly Gln

AmpR

agcaactcggtcgccgcatacactattctcagaatgacttggttgagtactcaccagtcacagaa 455
tcgttgagccagcggcgtatgtgataagagtcttactgaaccaactcatgagtggtcagtgtctt

Glu Gln Leu 90 Gly Arg Arg Ile His 95 Tyr Ser Gln Asn Asp 100 Leu Val Glu Tyr Ser 105 Pro Val Thr Glu

AmpR

aagcatcttacggatggcatgacagtaagagaattatgcagtgctgccataaccatgagtgataa 520
ttcgtagaatgcctaccgtactgtcattctcttaatacgtcacgacggtattggtactcactatt
110 115 120 125 130
Lys His Leu Thr Asp Gly Met Thr Val Arg Glu Leu Cys Ser Ala Ala Ile Thr Met Ser Asp Asn
AmpR

cactgcggccaacttacttctgacaacgatcggaggaccgaaggagctaaccgcttttttgcaca 585
gtgacgccggttgaatgaagactgttgctagcctcctggcttcctcgattggcgaaaaaacgtgt
135 140 145 150
Thr Ala Ala Asn Leu Leu Leu Thr Thr Ile Gly Gly Pro Lys Glu Leu Thr Ala Phe Leu His
AmpR

acatgggggatcatgtaactcgccttgatcgttgggaaccggagctgaatgaagccataccaaac 650
tgtaccccctagtacattgagcggaactagcaacccttggcctcgacttacttcggtatggtttg
155 160 165 170
Asn Met Gly Asp His Val Thr Arg Leu Asp Arg Trp Glu Pro Glu Leu Asn Glu Ala Ile Pro Asn
AmpR

gacgagcgtgacaccacgatgcctgtagcaatggcaacaacgttgcgcaaactattaactggcga 715
ctgctcgcactgtggtgctacggacatcgttaccgttgttgcaacgcgtttgataattgaccgct
175 180 185 190 195
Asp Glu Arg Asp Thr Thr Met Pro Val Ala Met Ala Thr Thr Leu Arg Lys Leu Leu Thr Gly Glu
AmpR

actacttactctagcttcccggcaacaattaatagactggatggaggcggataaagttgcaggac 780
tgatgaatgagatcgaagggccgttgttaattatctgacctacctccgcctatttcaacgtcctg
200 205 210 215
Leu Leu Thr Leu Ala Ser Arg Gln Gln Leu Ile Asp Trp Met Glu Ala Asp Lys Val Ala Gly
AmpR

cacttctgcgctcggcccttccggctggctggtttattgctgataaatctggagccggtgagcgt 845
gtgaagacgcgagccgggaaggccgaccgaccaaataacgactatttagacctcggccactcgca
220 225 230 235
Pro Leu Leu Arg Ser Ala Leu Pro Ala Gly Trp Phe Ile Ala Asp Lys Ser Gly Ala Gly Glu Arg
AmpR

gggtctcgcggtatcattgcagcactggggccagatggtaagccctcccgtatcgtagttatcta 910
cccagagcgccatagtaacgtcgtgaccccggtctaccattcgggagggcatagcatcaatagat
240 245 250 255 260
Gly Ser Arg Gly Ile Ile Ala Ala Leu Gly Pro Asp Gly Lys Pro Ser Arg Ile Val Val Ile Tyr
AmpR

AhdI

cacgacggggagtcaggcaactatggatgaacgaaatagacagatcgctgagataggtgcctcac 975
gtgctgcccctcagtccgttgatacctacttgctttatctgtctagcgactctatccacggagtg

265 270 275 280
Thr Thr Gly Ser Gln Ala Thr Met Asp Glu Arg Asn Arg Gln Ile Ala Glu Ile Gly Ala Ser

AmpR

tgattaagcattggtaactgtcagaccaagtttactcatatatactttagattgatttaaaactt 1040
actaattcgtaaccattgacagtctggttcaaatgagtatatatgaaatctaactaaattttgaa

285
Leu Ile Lys His Trp *

AmpR

catttttaatttaaaaggatctaggtgaagatcctttttgataatctcatgaccaaaatccctta 1105
gtaaaaattaaattttcctagatccacttctaggaaaaactattagagtactggttttagggaat

acgtgagttttcgttccactgagcgtcagaccccgtagaaaagatcaaaggatcttcttgagatc 1170
tgcactcaaaagcaaggtgactcgcagtctggggcatcttttctagtttcctagaagaactctag

ori

ctttttttctgcgcgtaatctgctgcttgcaaacaaaaaaaccaccgctaccagcggtggtttgt 1235
gaaaaaaagacgcgcattagacgacgaacgtttgtttttttggtggcgatggtcgccaccaaaca

ori

ttgccggatcaagagctaccaactctttttccgaaggtaactggcttcagcagagcgcagatacc 1300
aacggcctagttctcgatggttgagaaaaaggcttccattgaccgaagtcgtctcgcgtctatgg

ori

aaatactgtccttctagtgtagccgtagttaggccaccacttcaagaactctgtagcaccgccta 1365
tttatgacaggaagatcacatcggcatcaatccggtggtgaagttcttgagacatcgtggcggat

ori

catacctcgctctgctaatcctgttaccagtggctgctgccagtggcgataagtcgtgtcttacc 1430
gtatggagcgagacgattaggacaatggtcaccgacgacggtcaccgctattcagcacagaatgg

ori

gggttggactcaagacgatagttaccggataaggcgcagcggtcgggctgaacggggggttcgtg 1495
cccaacctgagttctgctatcaatggcctattccgcgtcgccagcccgacttgccccccaagcac

ori

cacacagcccagcttggagcgaacgacctacaccgaactgagatacctacagcgtgagctatgag 1560
gtgtgtcgggtcgaacctcgcttgctggatgtggcttgactctatggatgtcgcactcgatactc
ori

aaagcgccacgcttcccgaagggagaaaggcggacaggtatccggtaagcggcagggtcggaaca 1625
tttcgcggtgcgaagggcttccctctttccgcctgtccataggccattcgccgtcccagccttgt
ori

ggagagcgcacgagggagcttccagggggaaacgcctggtatctttatagtcctgtcgggtttcg 1690
cctctcgcgtgctccctcgaaggtccccctttgcggaccatagaaatatcaggacagcccaaagc
ori

ccacctctgacttgagcgtcgatttttgtgatgctcgtcaggggggcggagcctatggaaaaacg 1755
ggtggagactgaactcgcagctaaaaacactacgagcagtccccccgcctcggatacctttttgc
ori

PciI

ccagcaacgcggcctttttacggttcctggccttttgctggccttttgctcacatgttctttcct 1820
ggtcgttgcgccggaaaaatgccaaggaccggaaaacgaccggaaaacgagtgtacaagaaagga

gcgttatcccctgattctgtggataaccgtattaccgcctttgagtgagctgataccgctcgccg 1885
cgcaatagggggactaagacacctattggcataatggcggaaactcactcgactatggcgagcggc

cagccgaacgaccgagcgcagcgagtcagtgagcgaggaagcggaagagcgcccaatacgcaaac 1950
gtcggcttgctggctcgcgtcgctcagtcactcgctccttcgccttctcgcgggttatgcgtttg

cgcctctccccgcgcgttggccgattcattaatgcagctggcacgacaggtttcccgactggaaa 2015
gcggagaggggcgcgcaaccggctaagtaattacgtcgaccgtgctgtccaaagggctgacttt

gcgggcagtgagcgcaacgcaattaatgtgagttagctcactcattaggcaccccaggctttaca 2080
cgcccgtcactcgcgttgcgttaattacactcaatcgagtgagtaatccgtggggtccgaaatgt
CAP binding site
-35
lac promoter

ctttatgcttccggctcgtatgttgtgtggaattgtgagcggataacaatttcacacaggaaaca 2145
gaaatacgaaggccgagcatacaacacaccttaacactcgcctattgttaaagtgtgtcctttgt
-10
lac promoter
lac operator
M13 rev

```
gctatgaccatgattacgccaagcgcgcaattaaccctcactaaagggaacaaaagctggagctg   2210
cgatactggtactaatgcggttcgcgcgttaattgggagtgatttcccttgttttcgacctcgac
M13 rev                          T3 promoter

caagcttaatgtagtcttatgcaatactcttgtagtcttgcaacatggtaacgatgagttagcaa   2275
gttcgaattacatcagaatacgttatgagaacatcagaacgttgtaccattgctactcaatcgtt

catgccttacaaggagagaaaaagcaccgtgcatgccgattggtggaagtaaggtggtacgatcg   2340
gtacggaatgttcctctctttttcgtggcacgtacggctaaccaccttcattccaccatgctagc

tgccttattaggaaggcaacagacgggtctgacatggattggacgaaccactgaattgccgcatt   2405
acggaataatccttccgttgtctgcccagactgtacctaacctgcttggtgacttaacggcgtaa

gcagagatattgtatttaagtgcctagctcgatacaataaacgggtctctctggttagaccagat   2470
cgtctctataacataaattcacggatcgagctatgttatttgcccagagagaccaatctggtcta
                                          5' LTR (truncated)

ctgagcctgggagctctctggctaactagggaacccactgcttaagcctcaataaagcttgcctt   2535
gactcggaccctcgagagaccgattgatcccttgggtgacgaattcggagttatttcgaacggaa
5' LTR (truncated)

gagtgcttcaagtagtgtgtgcccgtctgttgtgtgactctggtaactagagatccctcagaccc   2600
ctcacgaagttcatcacacacgggcagacaacacactgagaccattgatctctagggagtctggg
5' LTR (truncated)

ttttagtcagtgtggaaaatctctagcagtggcgcccgaacagggacctgaaagcgaaagggaaa   2665
aaaatcagtcacaccttttagagatcgtcaccgcgggcttgtccctggactttcgctttcccttt
5' LTR (truncated)

ccagagctctctcgacgcaggactcggcttgctgaagcgcgcacggcaagaggcgaggggcggcg   2730
ggtctcgagagagctgcgtcctgagccgaacgacttcgcgcgtgccgttctccgctccccgccgc
HIV-1 Ψ

actggtgagtacgccaaaaattttgactagcggaggctagaaggagagagatgggtgcgagagcg   2795
tgaccactcatgcggtttttaaaactgatcgcctccgatcttcctctctctacccacgctctcgc
HIV-1 Ψ
```

NruI*

tcagtattaagcgggggagaattagatcgcgatgggaaaaaattcggttaaggccagggggaaag 2860
agtcataattcgccccctcttaatctagcgctacccttttttaagccaattccggtccccctttc

HIV-1 Ψ

aaaaaatataaattaaaacatatagtatgggcaagcagggagctagaacgattcgcagttaatcc 2925
tttttatatttaattttgtatatcatacccgttcgtccctcgatcttgctaagcgtcaattagg

tggcctgttagaaacatcagaaggctgtagacaaatactgggacagctacaaccatcccttcaga 2990
accggacaatctttgtagtcttccgacatctgtttatgaccctgtcgatgttggtagggaagtct

caggatcagaagaacttagatcattatataatacagtagcaaccctctattgtgtgcatcaaagg 3055
gtcctagtcttcttgaatctagtaatatattatgtcatcgttgggagataacacacgtagtttcc

atagagataaaagacaccaaggaagctttagacaagatagaggaagagcaaaacaaaagtaagac 3120
tatctctattttctgtggttccttcgaaatctgttctatctccttctcgttttgttttcattctg

NotI
EagI

caccgcacagcaagcggccgctgatcttcagacctggaggaggagatatgagggacaattggaga 3185
gtggcgtgtcgttcgccggcgactagaagtctggacctcctcctctatactccctgttaacctct

agtgaattatataaatataaagtagtaaaaattgaaccattaggagtagcacccaccaaggcaaa 3250
tcacttaatatatttatatttcatcatttttaacttggtaatcctcatcgtgggtggttccgttt

gagaagagtggtgcagagagaaaaaagagcagtgggaataggagctttgttccttgggttcttgg 3315
ctcttctcaccacgtctctcttttttctcgtcacccttatcctcgaaacaaggaacccaagaacc

RRE

gagcagcaggaagcactatgggcgcagcctcaatgacgctgacggtacaggccagacaattattg 3380
ctcgtcgtccttcgtgatacccgcgtcggagttactgcgactgccatgtccggtctgttaataac

RRE

BbvCI **BstAPI**

tctggtatagtgcagcagcagaacaatttgctgagggctattgaggcgcaacagcatctgttgca 3445
agaccatatcacgtcgtcgtcttgttaaacgactcccgataactccgcgttgtcgtagacaacgt

RRE

```
actcacagtctggggcatcaagcagctccaggcaagaatcctggctgtggaaagatacctaaagg   3510
tgagtgtcagaccccgtagttcgtcgaggtccgttcttaggaccgacacctttctatggatttcc
[RRE]

atcaacagctcctggggatttggggttgctctggaaaactcatttgcaccactgctgtgccttgg   3575
tagttgtcgaggacccctaaaccccaacgagaccttttgagtaaacgtggtgacgacacggaacc
[RRE]

                                BsaBI
aatgctagttggagtaataaatctctggaacagattggaatcacacgacctggatggagtgggac   3640
ttacgatcaacctcattatttagagaccttgtctaaccttagtgtgctggacctacctcaccctg

agagaaattaacaattacacaagcttaatacactccttaattgaagaatcgcaaaaccagcaaga   3705
tctctttaattgttaatgtgttcgaattatgtgaggaattaacttcttagcgttttggtcgttct

aaagaatgaacaagaattattggaattagataaatgggcaagtttgtggaattggtttaacataa   3770
tttcttacttgttcttaataaccttaatctatttacccgttcaaacaccttaaccaaattgtatt

caaattggctgtggtatataaaattattcataatgatagtaggaggcttggtaggtttaagaata   3835
gtttaaccgacaccatatattttaataagtattactatcatcctccgaaccatccaaattcttat

gtttttgctgtactttctatagtgaatagagttaggcagggatattcaccattatcgtttcagac   3900
caaaaacgacatgaaagatatcacttatctcaatccgtccctataagtggtaatagcaaagtctg

                    KflI
ccacctcccaaccccgaggggacccgacaggcccgaaggaatagaagaagaaggtggagagagag   3965
ggtggagggttggggctcccctgggctgtccgggcttccttatcttcttcttccacctctctctc

                                          HpaI
acagagacagatccattcgattagtgaacggatctcgacggttaacttttaaaagaaaagggggg   4030
tgtctctgtctaggtaagctaatcacttgcctagagctgccaattgaaaattttcttttcccccc
                                                [cPPT/CTS]

attggggggtacagtgcaggggaaagaatagtagacataatagcaacagacatacaaactaaaga   4095
taaccccccatgtcacgtcccctttcttatcatctgtattatcgttgtctgtatgtttgatttct
[cPPT/CTS]
```

ClaI
BspDI

attacaaaacaaattacaaaaattcaaaattttatcgataagcttgggagttccgcgttacata 4160
taatgttttgtttaatgtttttaagttttaaaatagctattcgaaccctcaaggcgcaatgtat

cPPT/CTS
CMV enhancer

acttacggtaaatggcccgcctggctgaccgcccaacgacccccgcccattgacgtcaataatga 4225
tgaatgccatttaccgggcggaccgactggcgggttgctgggggcgggtaactgcagttattact

CMV enhancer

cgtatgttcccatagtaacgccaatagggactttccattgacgtcaatgggtggagtatttacgg 4290
gcatacaagggtatcattgcggttatccctgaaaggtaactgcagttacccacctcataaatgcc

CMV enhancer

NdeI

taaactgcccacttggcagtacatcaagtgtatcatatgccaagtacgccccctattgacgtcaa 4355
atttgacgggtgaaccgtcatgtagttcacatagtatacggttcatgcgggggataactgcagtt

CMV enhancer

tgacggtaaatggcccgcctggcattatgcccagtacatgaccttatgggactttcctacttggc 4420
actgccatttaccgggcggaccgtaatacgggtcatgtactggaatacccctgaaaggatgaaccg

CMV enhancer

SnaBI

agtacatctacgtattagtcatcgctattaccatggtgatgcggttttggcagtacatcaatggg 4485
tcatgtagatgcataatcagtagcgataatggtaccactacgccaaaaccgtcatgtagttaccc

CMV enhancer
CMV promoter

cgtggatagcggtttgactcacggggatttccaagtctccaccccattgacgtcaatgggagttt 4550
gcacctatcgccaaactgagtgcccctaaaggttcagaggtggggtaactgcagttaccctcaaa

CMV promoter

gttttggcaccaaaatcaacgggactttccaaaatgtcgtaacaactccgccccattgacgcaaa 4615
caaaaccgtggttttagttgccctgaaaggttttacagcattgttgaggcggggtaactgcgttt

CMV promoter

tgggcggtaggcgtgtacggtgggaggtctatataagcagagctcgtttagtgaaccgtcagatc 4680
acccgccatccgcacatgccaccctccagatatattcgtctcgagcaaatcacttggcagtctag

CMV promoter

```
                                                 XbaI
gcctggagacgccatccacgctgtttgacctccatagaagacaccgactCTAGACACCATGGAA   4745
cggacctctgcggtaggtgcgacaaaactggaggtatcttctgtggctgaGATCTGTGGTACCTT
                                                          1
                                                          Met Glu
                                                          luciferase

GACGCCAAAAACATAAAGAAAGGCCCGGCGCCATTCTATCCGCTGGAAGATGGAACCGCTGGAGA   4810
CTGCGGTTTTTGTATTTCTTTCCGGGCCGCGGTAAGATAGGCGACCTTCTACCTTGGCGACCTCT
      5        10        15        20
Asp Ala Lys Asn Ile Lys Lys Gly Pro Ala Pro Phe Tyr Pro Leu Glu Asp Gly Thr Ala Gly Glu
luciferase

GCAACTGCATAAGGCTATGAAGAGATACGCCCTGGTTCCTGGAACAATTGCTTTTACAGATGCAC   4875
CGTTGACGTATTCCGATACTTCTCTATGCGGGACCAAGGACCTTGTTAACGAAAATGTCTACGTG
 25        30        35        40        45
Gln Leu His Lys Ala Met Lys Arg Tyr Ala Leu Val Pro Gly Thr Ile Ala Phe Thr Asp Ala
luciferase

                                 BstBI
ATATCGAGGTGGACATCACTTACGCTGAGTACTTCGAAATGTCCGTTCGGTTGGCAGAAGCTATG   4940
TATAGCTCCACCTGTAGTGAATGCGACTCATGAAGCTTTACAGGCAAGCCAACCGTCTTCGATAC
        50        55        60        65
His Ile Glu Val Asp Ile Thr Tyr Ala Glu Tyr Phe Glu Met Ser Val Arg Leu Ala Glu Ala Met
luciferase

AAACGATATGGGCTGAATACAAATCACAGAATCGTCGTATGCAGTGAAAACTCTCTTCAATTCTT   5005
TTTGCTATACCCGACTTATGTTTAGTGTCTTAGCAGCATACGTCACTTTTGAGAGAAGTTAAGAA
      70        75        80        85
Lys Arg Tyr Gly Leu Asn Thr Asn His Arg Ile Val Val Cys Ser Glu Asn Ser Leu Gln Phe Phe
luciferase

TATGCCGGTGTTGGGCGCGTTATTTATCGGAGTTGCAGTTGCGCCCGCGAACGACATTTATAATG   5070
ATACGGCCACAACCCGCGCAATAAATAGCCTCAACGTCAACGCGGGCGCTTGCTGTAAATATTAC
 90        95        100       105       110
Met Pro Val Leu Gly Ala Leu Phe Ile Gly Val Ala Val Ala Pro Ala Asn Asp Ile Tyr Asn
luciferase

AACGTGAATTGCTCAACAGTATGGGCATTTCGCAGCCTACCGTGGTGTTCGTTTCCAAAAAGGGG   5135
TTGCACTTAACGAGTTGTCATACCCGTAAAGCGTCGGATGGCACCACAAGCAAAGGTTTTTCCCC
        115       120       125       130
Glu Arg Glu Leu Leu Asn Ser Met Gly Ile Ser Gln Pro Thr Val Val Phe Val Ser Lys Lys Gly
luciferase
```

TTGCAAAAAATTTTGAACGTGCAAAAAAAGCTCCCAATCATCCAAAAAATTATTATCATGGATTC 5200
AACGTTTTTTAAAACTTGCACGTTTTTTTCGAGGGTTAGTAGGTTTTTTAATAATAGTACCTAAG
135 140 145 150
Leu Gln Lys Ile Leu Asn Val Gln Lys Lys Leu Pro Ile Ile Gln Lys Ile Ile Ile Met Asp Ser
luciferase

TAAAACGGATTACCAGGGATTTCAGTCGATGTACACGTTCGTCACATCTCATCTACCTCCCGGTT 5265
ATTTTGCCTAATGGTCCCTAAAGTCAGCTACATGTGCAAGCAGTGTAGAGTAGATGGAGGGCCAA
155 160 165 170 175
Lys Thr Asp Tyr Gln Gly Phe Gln Ser Met Tyr Thr Phe Val Thr Ser His Leu Pro Pro Gly
luciferase

BclI*
TTAATGAATACGATTTTGTGCCAGAGTCCTTCGATAGGGACAAGACAATTGCACTGATCATGAAC 5330
AATTACTTATGCTAAAACACGGTCTCAGGAAGCTATCCCTGTTCTGTTAACGTGACTAGTACTTG
180 185 190 195
Phe Asn Glu Tyr Asp Phe Val Pro Glu Ser Phe Asp Arg Asp Lys Thr Ile Ala Leu Ile Met Asn
luciferase

TCCTCTGGATCTACTGGTCTGCCTAAAGGTGTCGCTCTGCCTCATAGAACTGCCTGCGTGAGATT 5395
AGGAGACCTAGATGACCAGACGGATTTCCACAGCGAGACGGAGTATCTTGACGGACGCACTCTAA
200 205 210 215
Ser Ser Gly Ser Thr Gly Leu Pro Lys Gly Val Ala Leu Pro His Arg Thr Ala Cys Val Arg Phe
luciferase

CTCGCATGCCAGAGATCCTATTTTTGGCAATCAAATCATTCCGGATACTGCGATTTTAAGTGTTG 5460
GAGCGTACGGTCTCTAGGATAAAAACCGTTAGTTTAGTAAGGCCTATGACGCTAAAATTCACAAC
220 225 230 235 240
Ser His Ala Arg Asp Pro Ile Phe Gly Asn Gln Ile Ile Pro Asp Thr Ala Ile Leu Ser Val
luciferase

XcmI
TTCCATTCCATCACGGTTTTGGAATGTTTACTACACTCGGATATTTGATATGTGGATTTCGAGTC 5525
AAGGTAAGGTAGTGCCAAAACCTTACAAATGATGTGAGCCTATAAACTATACACCTAAAGCTCAG
245 250 255 260
Val Pro Phe His His Gly Phe Gly Met Phe Thr Thr Leu Gly Tyr Leu Ile Cys Gly Phe Arg Val
luciferase

GTCTTAATGTATAGATTTGAAGAAGCTGTTTCTGAGGAGCCTTCAGGATTACAAGATTCAAAG 5590
CAGAATTACATATCTAAACTTCTTCTCGACAAAGACTCCTCGGAAGTCCTAATGTTCTAAGTTTC
265 270 275 280
Val Leu Met Tyr Arg Phe Glu Glu Glu Leu Phe Leu Arg Ser Leu Gln Asp Tyr Lys Ile Gln Ser
luciferase

TGCGCTGCTGGTGCCAACCCTATTCTCCTTCTTCGCCAAAAGCACTCTGATTGACAAATACGATT 5655
ACGCGACGACCACGGTTGGGATAAGAGGAAGAAGCGGTTTTCGTGAGACTAACTGTTTATGCTAA

285 290 295 300 305
Ala Leu Leu Val Pro Thr Leu Phe Ser Phe Phe Ala Lys Ser Thr Leu Ile Asp Lys Tyr Asp
luciferase

TATCTAATTTACACGAAATTGCTTCTGGTGGCGCTCCCCTCTCTAAGGAAGTCGGGGAAGCGGTT 5720
ATAGATTAAATGTGCTTTAACGAAGACCACCGCGAGGGGAGAGATTCCTTCAGCCCCTTCGCCAA

310 315 320 325
Leu Ser Asn Leu His Glu Ile Ala Ser Gly Gly Ala Pro Leu Ser Lys Glu Val Gly Glu Ala Val
luciferase

GCCAAGAGGTTCCATCTGCCAGGTATCAGGCAAGGATATGGGCTCACTGAGACTACATCAGCTAT 5785
CGGTTCTCCAAGGTAGACGGTCCATAGTCCGTTCCTATACCCGAGTGACTCTGATGTAGTCGATA

330 335 340 345
Ala Lys Arg Phe His Leu Pro Gly Ile Arg Gln Gly Tyr Gly Leu Thr Glu Thr Thr Ser Ala Ile
luciferase

TCTGATTACACCCGAGGGGGATGATAAACCGGGCGCGGTCGGTAAAGTTGTTCCATTTTTTGAAG 5850
AGACTAATGTGGGCTCCCCCTACTATTTGGCCCGCGCCAGCCATTTCAACAAGGTAAAAAACTTC

350 355 360 365 370
Leu Ile Thr Pro Glu Gly Asp Asp Lys Pro Gly Ala Val Gly Lys Val Val Pro Phe Phe Glu
luciferase

CGAAGGTTGTGGATCTGGATACCGGGAAAACGCTGGGCGTTAATCAAAGAGGCGAACTGTGTGTG 5915
GCTTCCAACACCTAGACCTATGGCCCTTTTGCGACCCGCAATTAGTTTCTCCGCTTGACACACAC

375 380 385 390
Ala Lys Val Val Asp Leu Asp Thr Gly Lys Thr Leu Gly Val Asn Gln Arg Gly Glu Leu Cys Val
luciferase

AGAGGTCCTATGATTATGTCCGGTTATGTAAACAATCCGGAAGCGACCAACGCCTTGATTGACAA 5980
TCTCCAGGATACTAATACAGGCCAATACATTTGTTAGGCCTTCGCTGGTTGCGGAACTAACTGTT

395 400 405 410
Arg Gly Pro Met Ile Met Ser Gly Tyr Val Asn Asn Pro Glu Ala Thr Asn Ala Leu Ile Asp Lys
luciferase

GGATGGATGGCTACATTCTGGAGACATAGCTTACTGGGACGAAGACGAACACTTCTTCATCGTTG 6045
CCTACCTACCGATGTAAGACCTCTGTATCGAATGACCCTGCTTCTGCTTGTGAAGAAGTAGCAAC

415 420 425 430 435
Asp Gly Trp Leu His Ser Gly Asp Ile Ala Tyr Trp Asp Glu Asp Glu His Phe Phe Ile Val
luciferase

```
ACCGCCTGAAGTCTCTGATTAAGTACAAAGGCTATCAGGTGGCTCCCGCTGAATTGGAATCCATC   6110
TGGCGGACTTCAGAGACTAATTCATGTTTCCGATAGTCCACCGAGGGCGACTTAACCTTAGGTAG
           440                 445                 450                 455
Asp Arg Leu Lys Ser Leu Ile Lys Tyr Lys Gly Tyr Gln Val Ala Pro Ala Glu Leu Glu Ser Ile
luciferase
```

```
                   AarI                                   SgrAI
TTGCTCCAACACCCCAACATCTTCGACGCAGGTGTCGCAGGTCTTCCCGACGATGACGCCGGTGA   6175
AACGAGGTTGTGGGGTTGTAGAAGCTGCGTCCACAGCGTCCAGAAGGGCTGCTACTGCGGCCACT
       460                 465                 470                 475
Leu Leu Gln His Pro Asn Ile Phe Asp Ala Gly Val Ala Gly Leu Pro Asp Asp Asp Ala Gly Glu
luciferase
```

```
ACTTCCCGCCGCCGTTGTTGTTTTGGAGCACGGAAAGACGATGACGGAAAAAGAGATCGTGGATT   6240
TGAAGGGCGGCGGCAACAACAAAACCTCGTGCCTTTCTGCTACTGCCTTTTTCTCTAGCACCTAA
480                 485                 490                 495                 500
Leu Pro Ala Ala Val Val Val Leu Glu His Gly Lys Thr Met Thr Glu Lys Glu Ile Val Asp
luciferase
```

```
ACGTCGCCAGTCAAGTAACAACCGCGAAAAAGTTGCGCGGAGGAGTTGTGTTTGTGGACGAAGTA   6305
TGCAGCGGTCAGTTCATTGTTGGCGCTTTTTCAACGCGCCTCCTCAACACAAACACCTGCTTCAT
            505                 510                 515                 520
Tyr Val Ala Ser Gln Val Thr Thr Ala Lys Lys Leu Arg Gly Gly Val Val Phe Val Asp Glu Val
luciferase
```

```
CCGAAAGGTCTTACCGGAAAACTCGACGCAAGAAAAATCAGAGAGATCCTCATAAAGGCCAAGAA   6370
GGCTTTCCAGAATGGCCTTTTGAGCTGCGTTCTTTTTAGTCTCTCTAGGAGTATTTCCGGTTCTT
       525                 530                 535                 540
Pro Lys Gly Leu Thr Gly Lys Leu Asp Ala Arg Lys Ile Arg Glu Ile Leu Ile Lys Ala Lys Lys
luciferase
```

```
                    BamHI                                   BmgBI
GGGCGGAAAGATCGCCGTGGGATCCgagggcagaggaagtcttctaacatgcggtgacgtggagg   6435
CCCGCCTTTCTAGCGGCACCCTAGGctcccgtctccttcagaagattgtacgccactgcacctcc
545                 550                 140                 145                 150
Gly Gly Lys Ile Ala Val Gly Ser Glu Gly Arg Gly Ser Leu Leu Thr Cys Gly Asp Val Glu
luciferase                          T2A
```

```
                    SpeI
agaatcccggccctagtacaACTAGTGTGAGCAAGGGCGAGGAGCTGTTCACCGGGGTGGTGCCC   6500
tcttagggccgggatcatgtTGATCACACTCGTTCCCGCTCCTCGACAAGTGGCCCCACCACGGG
         155                                       5                  10
Glu Asn Pro Gly Pro Ser Thr Thr Ser Val Ser Lys Gly Glu Glu Leu Phe Thr Gly Val Val Pro
T2A                          1a
                             EGFP
```

ATCCTGGTCGAGCTGGACGGCGACGTAAACGGCCACAAGTTCAGCGTGTCCGGCGAGGGCGAGGG 6565
TAGGACCAGCTCGACCTGCCGCTGCATTTGCCGGTGTTCAAGTCGCACAGGCCGCTCCCGCTCCC

15 20 25 30 35
Ile Leu Val Glu Leu Asp Gly Asp Val Asn Gly His Lys Phe Ser Val Ser Gly Glu Gly Glu Gly

EGFP

CGATGCCACCTACGGCAAGCTGACCCTGAAGTTCATCTGCACCACCGGCAAGCTGCCCGTGCCCT 6630
GCTACGGTGGATGCCGTTCGACTGGGACTTCAAGTAGACGTGGTGGCCGTTCGACGGGCACGGGA

40 45 50 55
Asp Ala Thr Tyr Gly Lys Leu Thr Leu Lys Phe Ile Cys Thr Thr Gly Lys Leu Pro Val Pro

EGFP

GGCCCACCCTCGTGACCACCCTGACCTACGGCGTGCAGTGCTTCAGCCGCTACCCCGACCACATG 6695
CCGGGTGGGAGCACTGGTGGGACTGGATGCCGCACGTCACGAAGTCGGCGATGGGGCTGGTGTAC

60 65 70 75
Trp Pro Thr Leu Val Thr Thr Leu Thr Tyr Gly Val Gln Cys Phe Ser Arg Tyr Pro Asp His Met

EGFP

AAGCAGCACGACTTCTTCAAGTCCGCCATGCCCGAAGGCTACGTCCAGGAGCGCACCATCTTCTT 6760
TTCGTCGTGCTGAAGAAGTTCAGGCGGTACGGGCTTCCGATGCAGGTCCTCGCGTGGTAGAAGAA

80 85 90 95 100
Lys Gln His Asp Phe Phe Lys Ser Ala Met Pro Glu Gly Tyr Val Gln Glu Arg Thr Ile Phe Phe

EGFP

CAAGGACGACGGCAACTACAAGACCCGCGCCGAGGTGAAGTTCGAGGGCGACACCCTGGTGAACC 6825
GTTCCTGCTGCCGTTGATGTTCTGGGCGCGGCTCCACTTCAAGCTCCCGCTGTGGGACCACTTGG

105 110 115 120
Lys Asp Asp Gly Asn Tyr Lys Thr Arg Ala Glu Val Lys Phe Glu Gly Asp Thr Leu Val Asn

EGFP

GCATCGAGCTGAAGGGCATCGACTTCAAGGAGGACGGCAACATCCTGGGGCACAAGCTGGAGTAC 6890
CGTAGCTCGACTTCCCGTAGCTGAAGTTCCTCCTGCCGTTGTAGGACCCCGTGTTCGACCTCATG

125 130 135 140
Arg Ile Glu Leu Lys Gly Ile Asp Phe Lys Glu Asp Gly Asn Ile Leu Gly His Lys Leu Glu Tyr

EGFP

AACTACAACAGCCACAACGTCTATATCATGGCCGACAAGCAGAAGAACGGCATCAAGGTGAACTT 6955
TTGATGTTGTCGGTGTTGCAGATATAGTACCGGCTGTTCGTCTTCTTGCCGTAGTTCCACTTGAA

145 150 155 160 165
Asn Tyr Asn Ser His Asn Val Tyr Ile Met Ala Asp Lys Gln Lys Asn Gly Ile Lys Val Asn Phe

EGFP

```
CAAGATCCGCCACAACATCGAGGACGGCAGCGTGCAGCTCGCCGACCACTACCAGCAGAACACCC    7020
GTTCTAGGCGGTGTTGTAGCTCCTGCCGTCGCACGTCGAGCGGCTGGTGATGGTCGTCTTGTGGG
        170       175       180       185
Lys Ile Arg His Asn Ile Glu Asp Gly Ser Val Gln Leu Ala Asp His Tyr Gln Gln Asn Thr
EGFP >

CCATCGGCGACGGCCCCGTGCTGCTGCCCGACAACCACTACCTGAGCACCCAGTCCGCCCTGAGC    7085
GGTAGCCGCTGCCGGGGCACGACGACGGGCTGTTGGTGATGGACTCGTGGGTCAGGCGGGACTCG
        190       195       200       205
Pro Ile Gly Asp Gly Pro Val Leu Leu Pro Asp Asn His Tyr Leu Ser Thr Gln Ser Ala Leu Ser
EGFP >

AAAGACCCCAACGAGAAGCGCGATCACATGGTCCTGCTGGAGTTCGTGACCGCCGCCGGGATCAC    7150
TTTCTGGGGTTGCTCTTCGCGCTAGTGTACCAGGACGACCTCAAGCACTGGCGGCGGCCCTAGTG
        210       215       220       225       230
Lys Asp Pro Asn Glu Lys Arg Asp His Met Val Leu Leu Glu Phe Val Thr Ala Ala Gly Ile Thr
EGFP >

                        SalI
TCTCGGCATGGACGAGCTGTACAAGtaagtcgacaatcaacctctggattacaaaatttgtgaaa    7215
AGAGCCGTACCTGCTCGACATGTTCattcagctgttagttggagacctaatgttttaaacacttt
        235
Leu Gly Met Asp Glu Leu Tyr Lys *
EGFP                                WPRE

gattgactggtattcttaactatgttgctccttttacgctatgtggatacgctgctttaatgcct    7280
ctaactgaccataagaattgatacaacgaggaaaatgcgatacacctatgcgacgaaattacgga
WPRE
                                                   * His Arg

ttgtatcatgctattgcttcccgtatggctttcattttctcctccttgtataaatcctggttgct    7345
aacatagtacgataacgaagggcataccgaaagtaaaagaggaggaacatatttaggaccaacga
WPRE
Gln Ile Met Ser Asn Ser Gly Thr His Ser Glu Asn Glu Gly Gly Gln Ile Phe Gly Pro Gln Gln
(in frame with Factor Xa site)

gtctctttatgaggagttgtggcccgttgtcaggcaacgtggcgtggtgtgcactgtgtttgctg    7410
cagagaaatactcctcaacaccgggcaacagtccgttgcaccgcaccacacgtgacacaaacgac
WPRE
Arg Lys Ile Leu Leu Gln Pro Gly Asn Asp Pro Leu Thr Ala His His Ala Ser His Lys Ser
(in frame with Factor Xa site)
```

PflMI

acgcaacccccactggttgggcattgccaccacctgtcagctcctttccgggactttcgctttc 7475
tgcgttgggggtgaccaacccgtaacggtggtggacagtcgaggaaaggccctgaaagcgaaag

WPRE

Val Cys Gly Gly Ser Thr Pro Ala Asn Gly Gly Gly Thr Leu Glu Lys Gly Pro Ser Glu Ser Glu
(in frame with Factor Xa site)

cccctccctattgccacggcggaactcatcgccgcctgccttgcccgctgctggacaggggctcg 7540
ggggagggataacggtgccgccttgagtagcggcggacggaacgggcgacgacctgtccccgagc

WPRE

Gly Glu Arg Asn Gly Arg Arg Phe Glu Asp Gly Gly Ala Lys Gly Ala Ala Pro Cys Pro Ser Pro
(in frame with Factor Xa site)

gctgttgggcactgacaattccgtggtgttgtcggggaagctgacgtcctttccatggctgctcg 7605
cgacaacccgtgactgttaaggcaccacaacagccccttcgactgcaggaaaggtaccgacgagc

WPRE

Gln Gln Ala Ser Val Ile Gly His His Gln Arg Pro Leu Gln Arg Gly Lys Trp Pro Gln Glu
(in frame with Factor Xa site)

cctgtgttgccacctggattctgcgcgggacgtccttctgctacgtcccttcggccctcaatcca 7670
ggacacaacggtggacctaagacgcgccctgcaggaagacgatgcagggaagccgggagttaggt

WPRE

Gly Thr Asn Gly Gly Pro Asn Gln Ala Pro Arg Gly Glu Ala Val Asp Arg Arg Gly Glu Ile
(in frame with Factor Xa site)
1
Factor Xa site

SacII

gcggaccttccttcccgcggcctgctgccggctctgcggcctcttccgcgtcttcgccttcgccc 7735
cgcctggaaggaagggcgccggacgacggccgagacgccggagaaggcgcagaagcggaagcggg

WPRE

EcoRI Acc65I KpnI

tcagacgagtcggatctccctttgggccgcctccccgcctggaattcgagctcggtacctttaag 7800
agtctgctcagcctagagggaaacccggcggaggggcggaccttaagctcgagccatggaaattc

WPRE

accaatgacttacaaggcagctgtagatcttagccacttttaaaagaaaagggggggactggaag 7865
tggttactgaatgttccgtcgacatctagaatcggtgaaaaattttcttttccccccctgaccttc

3' LTR (ΔU3)

ggctaattcactcccaacgaagacaagatctgcttttgcttgtactgggtctctctggttagac 7930
ccgattaagtgagggttgcttctgttctagacgaaaacgaacatgacccagagagaccaatctg
3' LTR (ΔU3)

cagatctgagcctgggagctctctggctaactagggaacccactgcttaagcctcaataaagctt 7995
gtctagactcggaccctcgagagaccgattgatcccttgggtgacgaattcggagttatttcgaa
3' LTR (ΔU3)

gccttgagtgcttcaagtagtgtgtgcccgtctgttgtgtgactctggtaactagagatccctca 8060
cggaactcacgaagttcatcacacacgggcagacaacacactgagaccattgatctctagggagt
3' LTR (ΔU3)

gacccttttagtcagtgtggaaaatctctagcagtagtagttcatgtcatcttattattcagtat 8125
ctgggaaaatcagtcacaccttttagagatcgtcatcatcaagtacagtagaataataagtcata
3' LTR (ΔU3)

ttataacttgcaaagaaatgaatatcagagagtgagaggaacttgtttattgcagcttataatgg 8190
aatattgaacgtttctttacttatagtctctcactctccttgaacaaataacgtcgaatattacc
SV40 poly(A) signal

ttacaaataaagcaatagcatcacaaatttcacaaataaagcatttttttcactgcattctagtt 8255
aatgtttatttcgttatcgtagtgtttaaagtgtttatttcgtaaaaaaagtgacgtaagatcaa
SV40 poly(A) signal

gtggtttgtccaaactcatcaatgtatcttatcatgtctggctctagctatcccgcccctaactc 8320
caccaaacaggtttgagtagttacatagaatagtacagaccgagatcgatagggcggggattgag
SV40 poly(A) signal
SV40 ori

cgcccagttccgcccattctccgccccatggctgactaattttttttatttatgcagaggccgag 8385
gcgggtcaaggcgggtaagaggcggggtaccgactgattaaaaaaaataaatacgtctccggctc
SV40 ori

SfiI **StuI** **AvrII**

gccgcctcggcctctgagctattccagaagtagtgaggaggcttttttggaggcctaggcttttg 8450
cggcggagccggagactcgataaggtcttcatcactcctccgaaaaaacctccggatccgaaaac
SV40 ori

```
cgtcgagacgtacccaattcgccctatagtgagtcgtattacgcgcgctcactggccgtcgtttt    8515
gcagctctgcatgggttaagcgggatatcactcagcataatgcgcgcgagtgaccggcagcaaaa
                    T7 promoter                      M13 fwd

acaacgtcgtgactgggaaaaccctggcgttacccaacttaatcgccttgcagcacatccccctt    8580
tgttgcagcactgaccctttgggaccgcaatgggttgaattagcggaacgtcgtgtagggggaa
M13 fwd

tcgccagctggcgtaatagcgaagaggcccgcaccgatcgcccttcccaacagttgcgcagcctg    8645
agcggtcgaccgcattatcgcttctccgggcgtggctagcgggaagggttgtcaacgcgtcggac

aatggcgaatggcgcgacgcgccctgtagcggcgcattaagcgcggcgggtgtggtggttacgcg    8710
ttaccgcttaccgcgctgcgcgggacatcgccgcgtaattcgcgccgcccacaccaccaatgcgc
                              f1 ori

cagcgtgaccgctacacttgccagcgccctagcgcccgctcctttcgctttcttcccttcctttc    8775
gtcgcactggcgatgtgaacggtcgcgggatcgcgggcgaggaaagcgaaagaagggaaggaaag
                              f1 ori

tcgccacgttcgccggctttccccgtcaagctctaaatcgggggctccctttagggttccgattt    8840
agcggtgcaagcggccgaaaggggcagttcgagatttagcccccgagggaaatcccaaggctaaa
                              f1 ori

                                                      DraIII
agtgctttacggcacctcgaccccaaaaaacttgattagggtgatggttcacgtagtgggccatc    8905
tcacgaaatgccgtggagctggggttttttgaactaatcccactaccaagtgcatcacccggtag
                              f1 ori

gccctgatagacggtttttcgccctttgacgttggagtccacgttctttaatagtggactcttgt    8970
cgggactatctgccaaaaagcgggaaactgcaacctcaggtgcaagaaattatcacctgagaaca
                              f1 ori

tccaaactggaacaacactcaaccctatctcggtctattcttttgatttataagggattttgccg    9035
aggtttgaccttgttgtgagttgggatagagccagataagaaaactaaatattccctaaaacggc
                              f1 ori
```

atttcggcctattggttaaaaaatgagctgatttaacaaaaatttaacgcgaattttaacaaaat 9100
taaagccggataaccaatttttactcgactaaattgtttttaaattgcgcttaaaattgtttta
f1 ori

attaacgtttacaatttcc 3′ 9119
taattgcaaatgttaaagg 5′
f1 ori
